# Estimation of Cardiovascular Relative Pressure Using Virtual Work-Energy

**DOI:** 10.1038/s41598-018-37714-0

**Published:** 2019-02-04

**Authors:** David Marlevi, Bram Ruijsink, Maximilian Balmus, Desmond Dillon-Murphy, Daniel Fovargue, Kuberan Pushparajah, Cristóbal Bertoglio, Massimiliano Colarieti-Tosti, Matilda Larsson, Pablo Lamata, C. Alberto Figueroa, Reza Razavi, David A. Nordsletten

**Affiliations:** 1 Department of Biomedical Engineering and Health Systems, KTH Royal Institute of Technology, Stockholm, Sweden; 2 Department of Clinical Sciences, Karolinska Institutet, Stockholm, Sweden; 3 Division of Imaging Sciences and Biomedical Engineering, King’s College London, St Thomas’ Hospital, London, United Kingdom; 4 Department of Congenital Heart Disease, Evelina Children’s Hospital, London, United Kingdom; 5 Bernoulli Institute, University of Groningen, Groningen, The Netherlands; 6 Department of Clinical Science, Intervention and Technology (CLINTEC), Karolinska Institutet, Stockholm, Sweden; 7 Departments of Surgery and Biomedical Engineering, University of Michigan, Ann Arbor, USA; 8 Center for Mathematical Modeling, Universidad de Chile, Santiago, Chile

## Abstract

Many cardiovascular diseases lead to local increases in relative pressure, reflecting the higher costs of driving blood flow. The utility of this biomarker for stratifying the severity of disease has thus driven the development of methods to measure these relative pressures. While intravascular catheterisation remains the most direct measure, its invasiveness limits clinical application in many instances. Non-invasive Doppler ultrasound estimates have partially addressed this gap; however only provide relative pressure estimates for a range of constricted cardiovascular conditions. Here we introduce a non-invasive method that enables arbitrary interrogation of relative pressures throughout an imaged vascular structure, leveraging modern phase contrast magnetic resonance imaging, the virtual work-energy equations, and a *virtual field* to provide robust and accurate estimates. The versatility and accuracy of the method is verified in a set of complex patient-specific cardiovascular models, where relative pressures into previously inaccessible flow regions are assessed. The method is further validated within a cohort of congenital heart disease patients, providing a novel tool for probing relative pressures *in-vivo*.

## Introduction

Understanding the changes of relative pressure in segments of the cardiovascular system is critical for diagnosis and treatment planning in many cardiovascular diseases^1–3^. In aortic and pulmonary valve stenosis, the need for valve replacement is, in part, determined by the estimation of transvalvular relative pressure^1,4^. Similar procedures are following in patients with coarctation of the aorta, where possible intervention is inferred from the relative pressure drop through the aortic narrowing^5,6^. From clinical guidelines, the pressure drop in the left ventricular outflow tract is, furthermore, an established risk marker in hypertrophic cardiomyopathy patients^2^. These conditions provide a representative sample of a much broader range of conditions (coronary artery stenosis, aortic dissection, aneurysms, etc.) where differences in pressure facilitate diagnosis, treatment planning and prediction of disease progression. Clinical stratification through relative pressure has, consequently, become a routine component of cardiovascular medicine.

The clinical demand for assessing changes in hemodynamic load has driven the development of technologies that assess pressure behaviour *in-vivo*. To date, catheterisation remains the most direct measure, providing accurate real-time monitoring of pressure (or relative pressure)^7,8^. Despite its capacity to map the cost of driving flow, the risks associated with invasive catheterisation limit its practical utility as a general-purpose diagnostic tool^9–11^. Current clinical guidelines instead leverage non-invasive image-based estimates, where acquired velocity measures are linked to relative pressure using fluid mechanics principles^1,7^. Across a range of cardiovascular disease^1,3,12,13^, Doppler echocardiography is by far the most commonly used surrogate measure, linking peak velocity measurements to relative pressure through a simplified form of Bernoulli’s equation^1^. While providing an effective diagnostic indicator for these conditions, it is well known that relative pressure estimates from Doppler often vary from invasive measures^14–16^, and further vary based on the operator^17,18^. Doppler estimates are additionally restricted to a portion of cardiovascular conditions where the superficial access of the probe is sufficient for measuring flow. Consequently, the extension of Doppler estimates to the broader class of cardiovascular disease with complex morphology remains limited.

More recent advances in medical imaging – providing full-field velocity measurements throughout the cardiovascular system – are broadening the scope for non-invasive pressure assessment. Measurement of cardiovascular flow regions can now be acquired using 3D phase contrast magnetic resonance imaging (or so-called 4D flow MRI)^19^ as well as 3D Doppler ultrasound^20^. Availability of flow data has largely determined the way relative pressures are estimated, with simplified Bernoulli providing the best approximate relative pressure based on a single maximal velocity measurement. The advent of full-field measures, however, has enabled the development of new techniques capable of leveraging the full strength of the Navier-Stokes equations (NSE) – commonly employed to understand cardiovascular flow^21–23^ – with fewer underlying assumptions. Measures using the pressure poisson equation (PPE) have been suggested for pressure estimation^24,25^, showing some promising results^26,27^. However, such models are characterized by strong biases and sensitivity to the defined flow domain^16,28^, as well as decreasing accuracy when applied on regions of restricted, narrow flow^29,30^. Forms including turbulent energy have also been suggested^31^. In recent work, we outlined a formulation that evaluates relative pressure using the work-energy form of NSE^32^. While demonstrating improved accuracy, the work-energy relative pressure (WERP) estimates were limited to single vessel compartments and regimes of large flow magnitudes. Thus, while these techniques demonstrated validity in specific scenarios, methods capable of generally estimating *in-vivo* relative pressure, through complex cardiovascular anatomy and throughout the cardiac cycle, remain elusive.

In this paper, we present a novel technique that extends the WERP formulation^16,32^, and our subsequent work in Bertoglio *et al*.^29^, using the concept of virtual fields. Through the creation of an auxiliary flow field we consider the virtual work-energy relative pressure (*ν*WERP) required to drive the imaged flow between arbitrarily selected vascular cross-sections. The method is able to probe relative pressure differences in previously inaccessible sections, with an accuracy above that of routinely employed techniques. In the following, we demonstrate our method using image-based data and evaluate its capacity to deal with flow complexities encountered *in-vivo*. The image-based *ν*WERP estimates are evaluated for accuracy, noise sensitivity and spatiotemporal behaviour in a set of *in-silico* imaging datasets, before being validated *in-vivo* by comparing *ν*WERP estimates derived from 4D flow images against invasive catheter measurements - all illustrating the method’s capacity to estimate relative pressure throughout entire vascular spaces over time.

## Results

### Virtually probing hemodynamic flow to compute relative pressure

**Figure 1:**
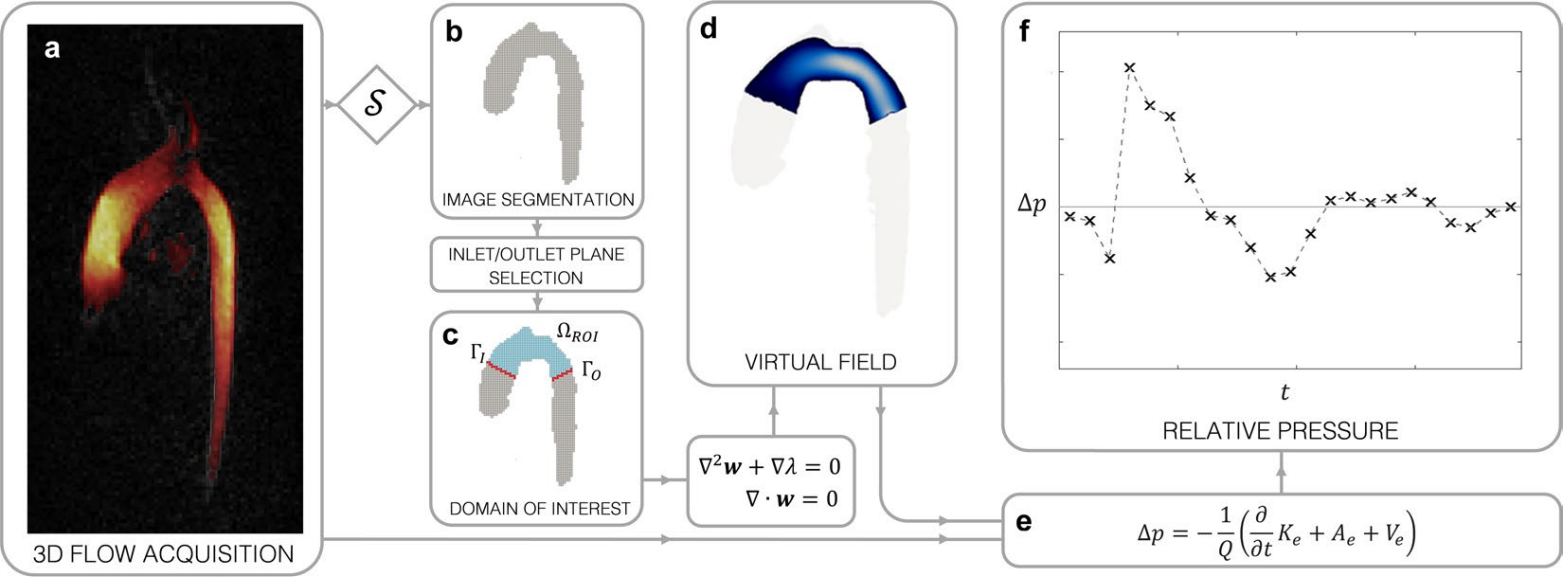
Overview of *ν*WERP estimation from 4D flow data. **(a)** Acquisition of 4D flow with velocity magnitude (>1/4^th^ maximum velocity) highlighted by the red-to-yellow color scale. **(b)** Segmentation of the primary flow domain. **(c)** Manual selection of inlet and outlet planes, Γ_*I*_ and Γ_*O*_, for the relative pressure estimation. **(d)** Using segmentation, the domain of interest, Ω_*ROI*_, and inlet/outlet planes, a separate Stokes flow boundary value problem is solved to create the virtual field ***w***. **(e)** The virtual work-energy of the field ***w*** is combined with the acquired velocity field ***v*** to compute the relative pressure. **(f)** Final relative pressure between inlet and outlet planes over time.

The complete theory and process for computing relative pressure using the virtual Work-Energy Relative Pressure (*ν*WERP) formulation is described in detail in the Online Methods, and in Fig. 1. Briefly, from an acquired 4D flow field, the method requires segmentation of the main vascular flow compartment. Inlet and outlet planes are then selected between which relative pressures are to be estimated. A virtual field to interrogate the anatomical region of interest is computed based on the solution to a Stokes flow problem, isolating the relative pressure of interest. Combining the acquired flow and virtual fields, *ν*WERP evaluates virtual work-energy equations to identify relative pressure over time. While *ν*WERP is not inherently connected to any specific imaging modality, here we focus on integration of MRI-based 4D flow. The method could however easily be adapted to other full-field imaging modalities such as refined 3D ultrasound^20^ or contrast-enhanced computed tomography^33^.

### *In-silico* evaluation in cases of aortic coarctation and aortic dissection

**Figure 2:**
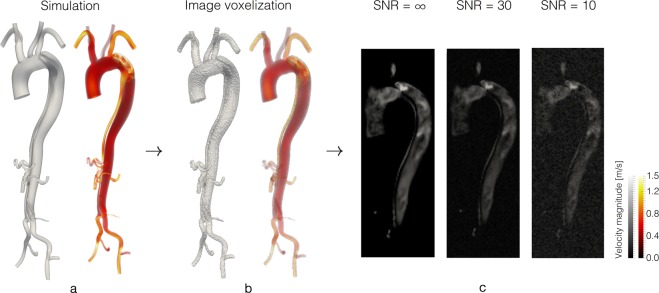
Generation of *in-silico* image data from patient-specific flow simulations. **(a)** Patient-specific simulation of an acute type B dissected aorta (left) with corresponding mid-systolic (*t* = 0.17 s) velocity magnitude (right). **(b)** Simulated patient-specific geometry resampled onto a uniform image voxel grid of 2 mm^3^ (left) together with the corresponding flow field (right). **(c)** Coronal cut through the aortic arch in the *in-silico* image data without noise (SNR = ∞, left), with low-noise (SNR = 30, middle), and with high-noise (SNR = 10, right).

To evaluate the *ν*WERP method in a set of challenging realistic flow scenarios, a set of personalised *in-silico* tests were performed using Computational Fluid Dynamics (CFD) (for a full description of the models used, see Online Methods). Analysis using *in-silico* image data provides a powerful test-bed for new techniques, as velocity and pressure information can be defined at high spatiotemporal resolution throughout the entire vasculature, giving a clear comparative ground truth. Two pathological conditions are considered in this work: a model of a coarctation of the aorta (CoA)^34^ and a model of an acute type B aortic dissection (AAD)^35^. Both reflect pathological cases where relative pressure is of direct diagnostic importance^6,36^. *In-silico* results were sampled using spatiotemporal resolution typical of 4D flow data (2 mm^3^ voxel size images at 22 and 32 phases over the cardiac cycle for CoA and AAD, respectively) as illustrated in Fig. 2. Effects of realistic signal-to-noise (SNR) levels were evaluated by amending the sampled *in-silico* results with 100 different noise fields at low- (SNR = 30) and high-noise (SNR = 10), respectively. With the *in-silico* image data generated, the flow fields were fed into the *ν*WERP work-flow (Fig. 1) and relative pressures were estimated between sets of vascular planes.

**Figure 3:**
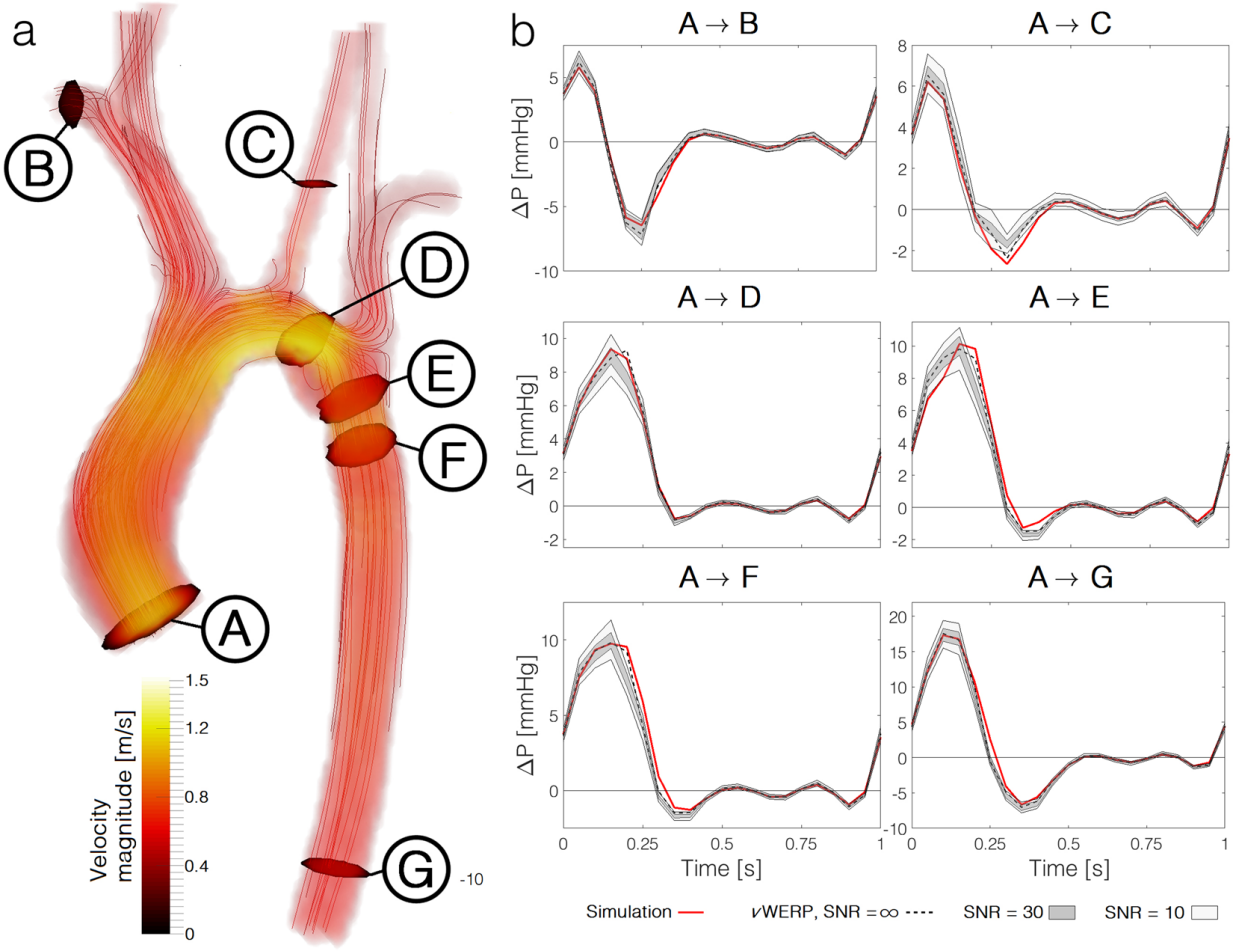
*ν*WERP relative pressure estimation in a patient-specific coarcted aorta. **(a)** Visualisation of velocity magnitude with streamlines, along with all selected inlet and outlet planes indicated by A – G. **(b)** Relative pressure estimations from aortic inlet (A) to all selected outlet planes (B – G) as a function of time. True pressure from simulations is given by the red continuous line, *ν*WERP estimates given by the dashed black, dark gray, and light gray fields, for SNR = ∞/30/10, respectively.

Figure 3 shows the CoA results, illustrating planes between which relative pressures were estimated and providing graphs comparing *ν*WERP estimates to the simulated (CFD) ground-truth. In general, a distinct correspondence can be seen between *ν*WERP and true (CFD-based) relative pressure, with errors consistently below 20% for all evaluated planes and SNR, (peak systolic error < 1.0 mmHg), with a mean error of 10% at SNR = ∞ (averaged over the entire cardiac cycle). In particular, the method shows the ability to accurately probe the relative pressure between multiple sites, with *ν*WERP estimates showing no evidence of bias due to vascular branching. A slight increase in error (mean error average 13/18/18% at SNR = ∞/30/10) can be seen into the left common carotid artery (plane C in Fig. 3), potentially due to the smaller mean diameter of 6 cm (~3 voxels).

*ν*WERP estimates demonstrate robustness to noise – important for clinical use – showing only minor increases in mean error (13/15% at SNR = 30/10). For all noise-levels the *ν*WERP estimates consistently follow the general behaviour of the simulated pressure through the cardiac cycle. Focusing on the clinically important coarctation (D → E, Fig. 3), *ν*WERP estimates give mean errors of below 16/18% for SNR = 30/10, indicating the potential value of this technique to compute quantities of important clinical value.

**Figure 4:**
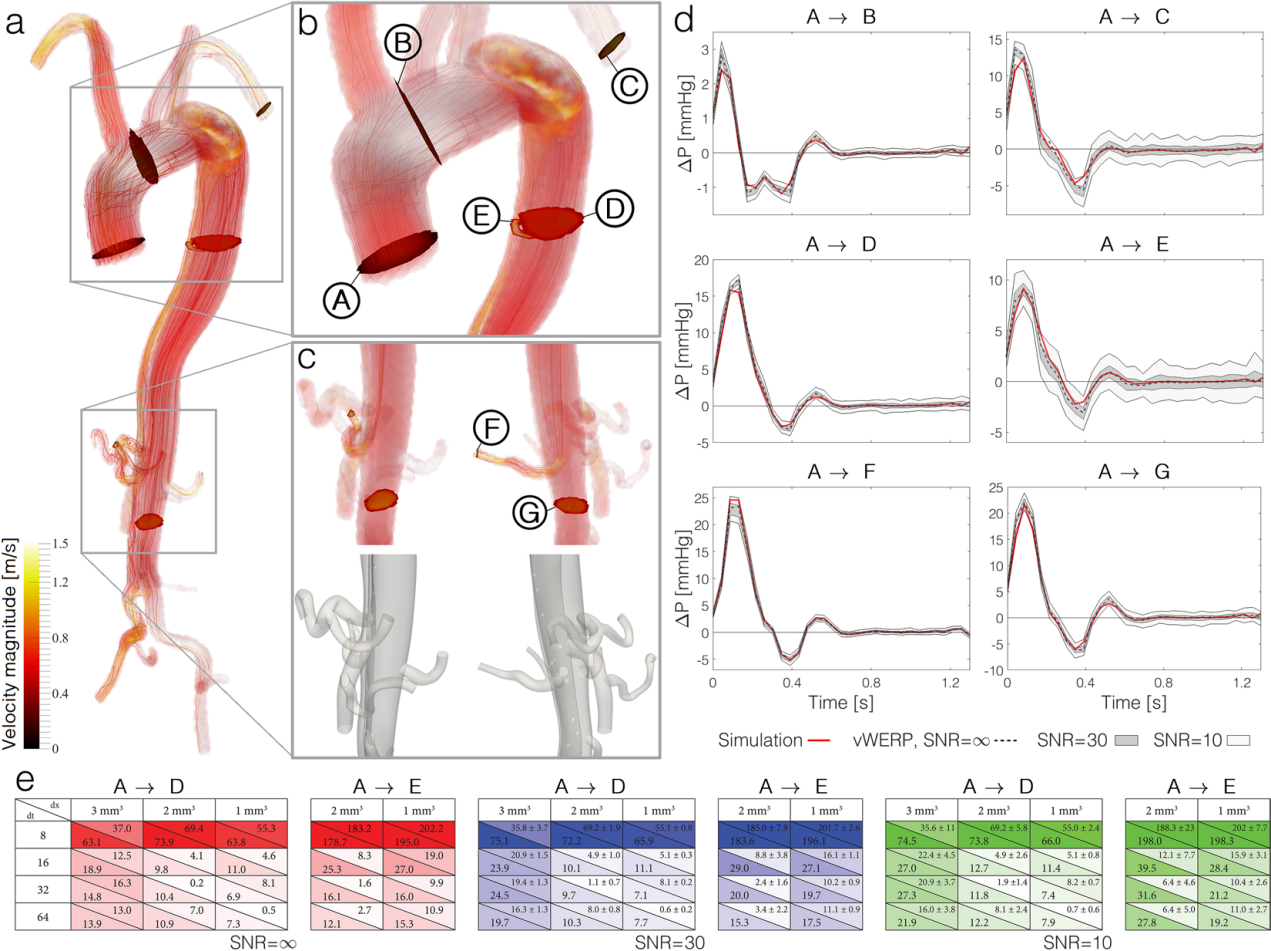
*ν*WERP relative pressure estimation in a patient-specific dissected aorta, including spatiotemporal convergence analysis. **(a)** Visualisation of velocity magnitude with streamlines, along with all selected inlet and outlet planes. **(b)** Close-up visualisation of aortic arch, showing inlet (A), upper descending false lumen (D) and upper descending true lumen (E) planes. **(c)** Close-up visualisation of outlet planes at the renal artery connecting both true and false lumen (F), as well as at the distal part of the true lumen (G). Views are provided for the same orientation as (a) (left) and as well as rotated 90 degrees (right) **(d)** Relative pressure estimation from aortic inlet (A) to all selected outlet planes (B – G) as a function of time. True pressure from simulations is given by the red continuous line, *ν*WERP estimates given by the dashed black, dark gray, and light gray fields, for SNR = ∞/30/10, respectively. **(e)** Analysis of spatiotemporal resolution from the aortic inlet (A) to false (D) and true lumen (E). Note that the analysis is only performed when the selected spatial image sampling is able to resolve the anatomy. Data for SNR = ∞/30/10 is given in red/blue/green, respectively. Each table entry presents the mean error (lower left) and the error at peak relative pressure with standard deviation (upper right).

Figure 4 similarly presents results for a selection of anatomical planes in the AAD model. In this instance, the flow is significantly impacted by the septum separating true and false lumen as well as the numerous connecting tears linking flow between the lumina. However, with the use of a virtual field, *ν*WERP was able to isolate the relative pressure of interest (a visualisation of the virtual field into true and false lumina is given in Supplementary Fig. 3). As with the CoA, *ν*WERP illustrates the capacity to probe relative pressure between any of the selected points in the dissected aorta, with a mean error of below 14/16/21% for all evaluated regions at SNR = ∞/30/10. With noise, narrower regions show a slight deterioration in accuracy such as seen for the right subclavian artery (plane C, lumen diameter ~4 voxels) and the descending aortic part of the true lumen (plane E, lumen diameter ~3 voxels), showing mean errors of 23/24/31% and 16/20/32% at SNR = ∞/30/10, respectively. However, *ν*WERP estimates still follow the temporal behaviour of the true (CFD-based) relative pressure.

A particularly complex case is provided for plane F: here, the right renal artery is fed by the true lumen, before continuing into the false lumen and the ascending thoracic aorta through connecting tears. Given sufficient spatial sampling to capture the narrow anatomy (in this case 1 mm^3^), *ν*WERP shows the ability to estimate relative pressure with a mean error of 12%. This again exemplifies the strength of the proposed approach, and underlines the versatility of using virtual fields to probe relative pressures even in highly complex cardiovascular morphologies.

For the above evaluated *in-silico* patient-specific cases, the application of conventional flow based relative pressure estimates become problematic, specifically since the test cases considered expand beyond the usual intended use of these methods. The routinely used Bernoulli estimate originates from a singular measure of peak velocity, and similarly the extension into the unsteady Bernoulli^37^ neglects a large portion of the flow field and is not optimised for traces into vasculatures of turbulent or low flow. Using work-energy estimates without the use of a virtual field^32^ also deteriorate with vascular branching or low flow regimes. For these reasons, the above methods perform poorly for both CoA and AAD, with none of the evaluated relative pressures successfully assessed. Comparing with *ν*WERP estimates into the true lumen of the AAD (A → E, Fig. 4) render mean errors of 100%, 88%, and > 200% for simplified Bernoulli, unsteady Bernoulli and conventional work-energy estimates, respectively (a complete numerical comparison is given in Supplementary Table 3). With more refined approaches such as the PPE showing reduced accuracy even in simplified analytical setups^29^, the added value of the proposed *ν*WERP approach is highlighted, with a gained ability to probe relative pressure in regions inaccessible by other proposed methods.

### *In-silico* evaluation of image resolution and SNR

A core practical concern for image-driven techniques is their dependence on spatiotemporal resolution and image quality. Using the cardiovascular simulations, where image spatial and temporal resolution can be freely adapted, it is feasible to explore the impact of resolution and noise on *ν*WERP performance. For this analysis, two regions spanning from ascending aorta to descending true and false lumen of the AAD (A → D and A → E, Fig. 4) were selected, with temporal and spatial sampling within clinical acquired image ranges (for details of the model generation, see Online Methods).

In general, *ν*WERP performance improves with increasing spatiotemporal sampling, as seen in Fig. 4e. For the mean error, this behaviour is seen for both the wide false and the narrow true lumen, respectively, despite the significant flow differences. Considering image noise, the results indicate that *ν*WERP can assess relative pressure in all evaluated noise scenarios with regards to both peak and mean relative pressure, even if image noise exhibits a more pronounced effect on the estimates into the narrower true lumen (at 2 mm^3^ and 32 time frames, mean error is 16/20/32% in the true lumen, compared to 10/10/12% in the wider false lumen at SNR = ∞/30/10). With a reduction in the number of voxels, increasing boundary layer effects as well as singular noise will influence the physics of the investigated flow and impact *ν*WERP estimation accuracy. The sought estimate accuracy will thus require consideration of both the image acquisitions settings, as well as the vasculature of interest. Regardless, *ν*WERP still shows reasonable behaviour in the narrow true lumen when using routine imaging settings (2 mm^3^ voxel size and 32 temporal phases), where the peak relative pressure was estimated with an accuracy of 2 ± 2/6 ± 5% at SNR = 30/10.

Analyses at the noise-free state (SNR = ∞) indicate a slightly higher dependence on temporal sampling, where a twofold increase in temporal sampling renders an average decrease in mean error of 32%, whereas a similar increase in spatial sampling gives an average decrease in error of 19%. However, the effect seems slightly reversed when image noise is added, where a twofold increase in spatial and temporal sampling renders a decrease in mean error of 34/39% and 31/31% at SNR = 30/10, respectively. For the peak error the effect of increased spatial does not appear as clear, even though temporal refinements seems to have a defined effect at all noise levels (twofold temporal refinement rendering a decrease in peak error of 26/30/32% and 39/40/43% at SNR = ∞/30/10 for false and true lumen, respectively). Consequently, the appropriate choice of spatiotemporal image sampling will be dependent on the physical nature of the investigated vasculature and demand for accuracy; narrow vessels require increased spatial sampling, whereas transient cardiac events (such as those during ventricular ejection in early systole) require increased temporal sampling.

An additional analytical spatiotemporal convergence analysis is provided in Supplementary Material.

### *In-vivo* validation with invasive cardiac catheterisation

To establish its clinical applicability, we compared *ν*WERP estimates against gold-standard invasive pressure measurements acquired *in-vivo* during combined cardiac catheterisation and cardiac MRI (XMR). 4D flow MRI (voxel size ~2 mm^3^, 22 time samples) was collected in a cohort of five anaesthetised adolescent patients with complex congenital heart disease (for patient characteristics, see Supplementary Table 1). During the same XMR session, absolute invasive pressures were recorded through catheterisation at ascending and mid-descending or diaphragm level of the aorta, respectively (positions registered using fluoroscopy). For a complete description of data preparation, see Online Methods. All patients included in this study participated under informed consent, with data collection and study approved by the Regional Ethics Committee, South East London, United Kingdom (REC, 10/H0802/65). Additionally, all methods and experiments were performed in accordance with relevant guidelines and regulations.

**Figure 5:**
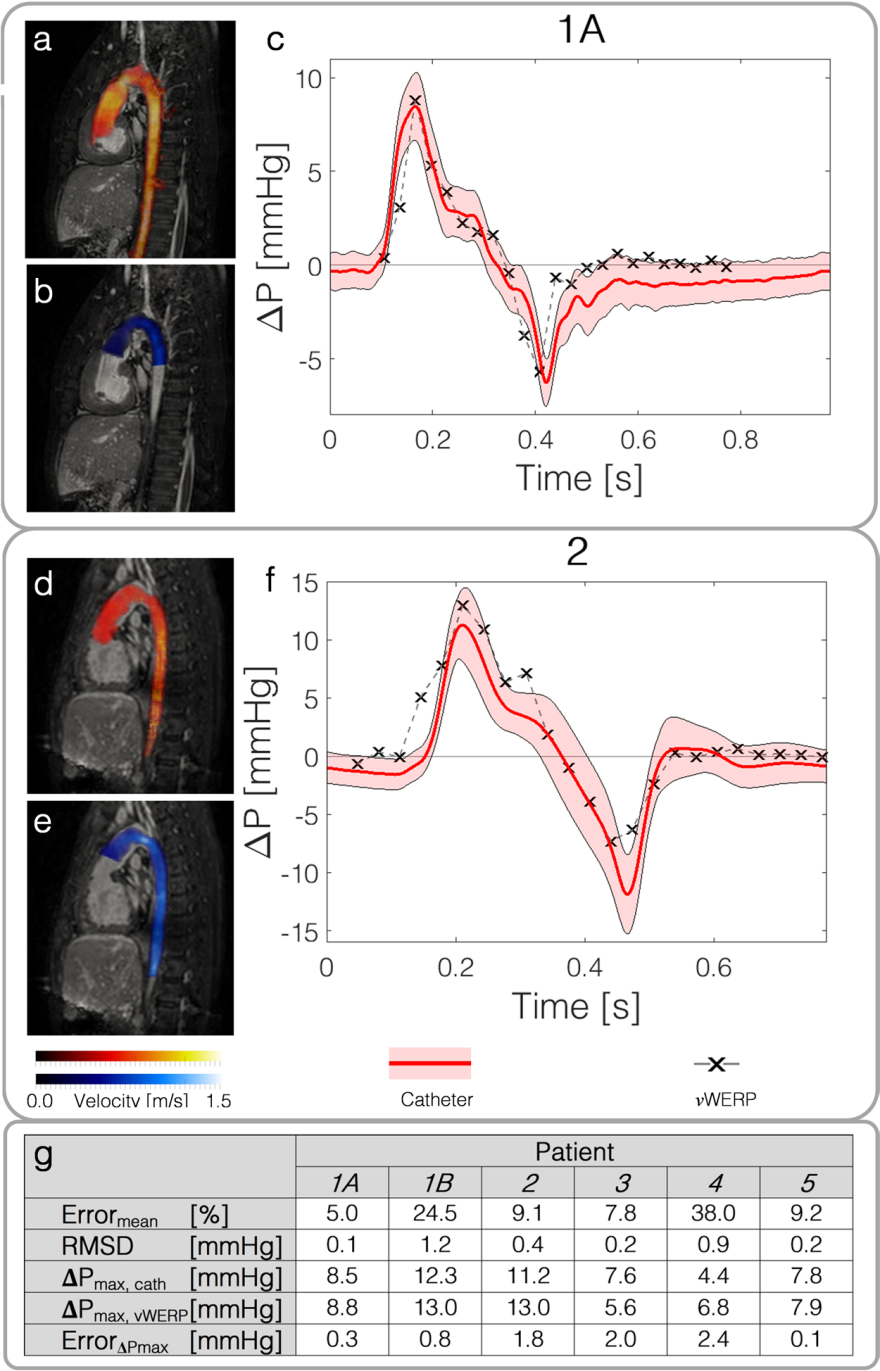
Validation of *ν*WERP against invasive catheter measurements in the aortic arch of a cohort of complex congenital heart disease patients. **(a**,**d)** Acquired flow field patients 1 A and 2. **(b**,**e)** Computed virtual field, isolating the domain of interest over which relative pressure estimates are derived. **(c**,**f)** Relative pressure estimation from inlet to the diaphragm level of the descending aorta by catheterisation (red continuous line with distribution) and *ν*WERP (black glyphs with dashed grey line). **(g)** Table summarising the results for the entire cohort, indicating mean error, root-mean square difference (RMSD), peak relative pressure by catheterisation and *ν*WERP, respectively, as well as the corresponding absolute error.

Results for all six subjects are presented in Fig. 5. On average, *ν*WERP could capture the temporal shift in relative pressure, with estimates corresponding well to gold-standard catheter measurements (for a graphical comparison of all investigated patients, see Supplementary Fig. 4). A mean error of 16 ± 13% is reported for all subjects, corresponding to an absolute mean error < 1.0 mmHg. The error varied slightly over time, with the initial systolic pressure peak captured with defined accuracy in all patients (mean error < 18%, corresponding to an accuracy within 1.2 mmHg). The return from the systolic pressure peak is also well captured by *ν*WERP as seen in both Fig. 5 and Supplementary Fig. 4. With the relative pressure in general, and the peak relative pressure specifically acting as an important clinical biomarker^1,3^, *ν*WERP shows direct utility for clinical diagnostics. Additionally, with this accuracy reported over the aortic arch where several vessel branches are present, as well as into the narrow adolescent descending aortas (in average ~5 voxels over the vessel cross-section), *ν*WERP enables recognised biomarkers to be applied in vascular regions where it has previously not been used, following limitations in the routinely and previously proposed methods.

Comparing alternative methods, these again perform with varying accuracy, following the unconventional application field for which none of the methods are optimised. Using simplified Bernoulli errors in maximum relative pressure of >100% were found, with the accuracy significantly deteriorating due to the simplifications imposed in the hemodynamic description of the flow. For the extension to Unsteady Bernoulli, the accuracy increases (error at peak systole of 67% or 6.2 mmHg), although it produces errors of above 100% in singular subjects due to the assumptions made on the assessed flow. With regards to the work-energy based approach without the virtual field mean errors in relative pressure of >300% were found, again following the branched anatomy as well as low diastolic flows. Lastly, the unsuitability of the PPE method to deal with the narrow adolescent lumina further highlights the novelty of the proposed *ν*WERP method, which represents a definite improvement for hemodynamic pressure estimations and enables the probing of relative pressure in a range of vascular segments accessible by image-based 4D flow acquisition.

### Cumulative analysis of *ν*WERP estimates of relative pressure

**Figure 6:**
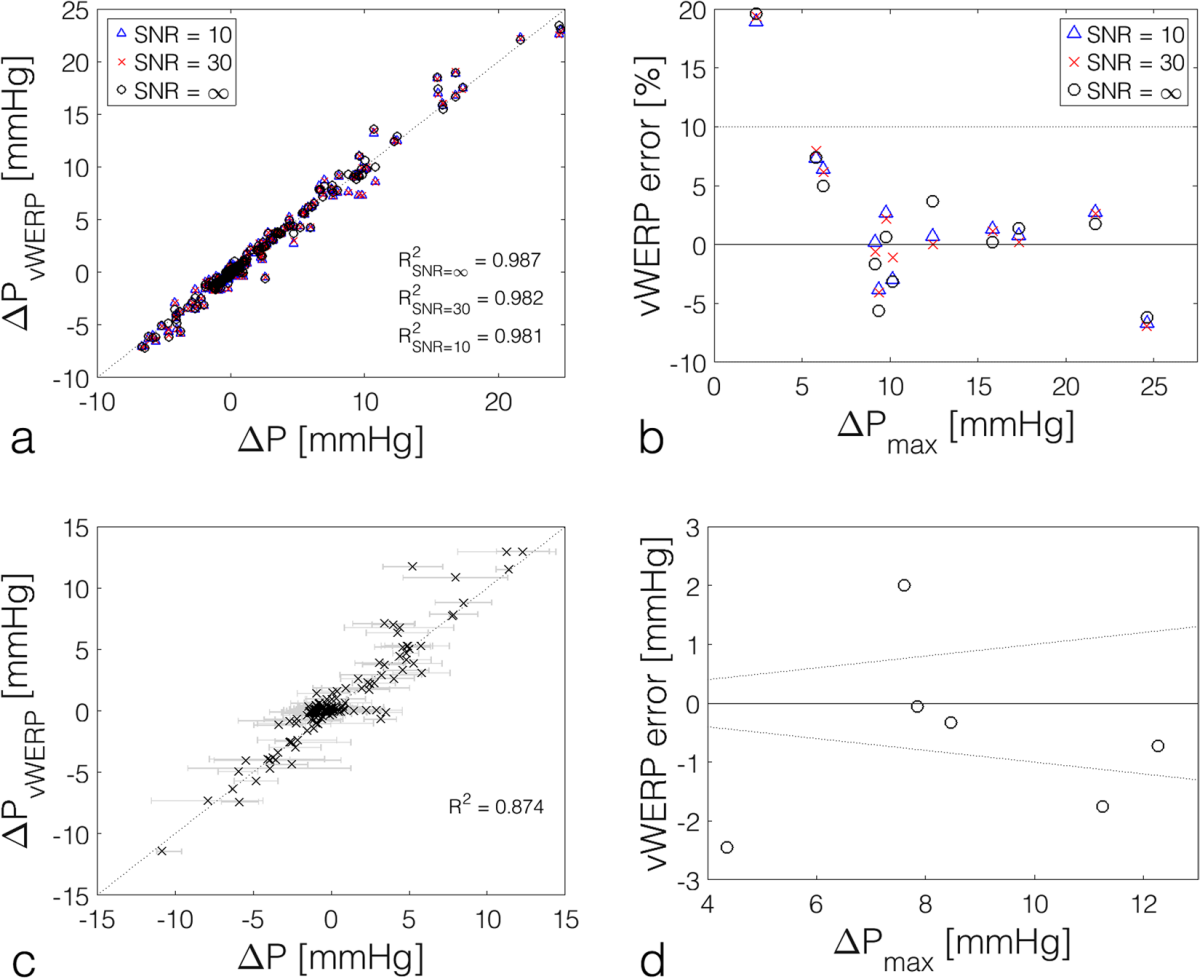
Cumulative analysis of *ν*WERP estimates in both *in-silico* and *in-vivo* evaluations. **(a)** Relative pressure estimations for patient-specific coarcted and dissected aorta, respectively, comparing *ν*WERP (y-axis) to simulated data (x-axis). **(b)** Relative error of *ν*WERP estimates at peak relative pressure for all evaluated inlet/outlet planes in patient-specific coarcted and dissected aortas. Data for SNR = ∞/30/10 is given by black circles, red crosses, and blue triangles, respectively. **(c)** Relative pressure estimation for the validation study, comparing *ν*WERP estimates (y-axis) to catheter measurements (x-axis). The distribution of the catheter data is given by gray bars, with *ν*WERP estimates superimposed by black crosses. **(d)** Absolute error of *ν*WERP estimates at peak relative pressure for the entire validation study cohort. Data for estimates against the mean of all catheter measurements is given by the black circles, and relative errors of ±10% are given by the dashed black lines.

Figure 6 presents the estimated relative pressure compared to true values for all *in- silico* and *in-vivo* scenarios (details on error methodology are found in the Online Methods). Figure 6a shows results from *in-silico* image data over all evaluated cases seen in Figs 3 and 4 (2 mm^3^ and 22/32 time frames). Only minor deviations from an ideal 1:1 relationship can be seen, with average linear regression slopes of *m* = 0.99/0.98/0.98 for SNR = ∞/30/10. Comparing peak relative pressures, results again illustrate consistency, with a slight overestimation (peak relative pressure errors of 1.9/2.2/2.3% for SNR = ∞/30/10). From the results in the tables of Fig. 4e, increased spatiotemporal sampling of the data could potentially improve these overestimations.

Examining the *in-vivo* data, the deviation between *ν*WERP and the catheter measurements shows more variability likely due to catheter acquisition issues^38^, inherent patient variability, and imaging artefacts. However, the correlation between *ν*WERP and the catheter measures remains strong, with an average slope of *m* = 0.87 (Fig. 6c), representing a slight underestimation in relative pressure. Isolating the peak relative pressure (Fig. 6d), the absolute and mean error does seem to comply with a slight underestimation (0.6 ± 1.5 mmHg or 8 ± 26%). Importantly, considering the improvement in performance compared to other methods, and judging from the novelty in accessible regions of which the above *ν*WERP validation is performed, the proposed approach represents a novel method for improved hemodynamic diagnostics in cardiovascular applications.

## Discussion

In this work, we have presented a work-energy based method (*ν*WERP) to derive relative pressure between any given vascular segment within an acquired flow field. This versatility is provided by the use of a *virtual field* to define the vascular region of interest, achieving accurate assessments of relative pressure. *ν*WERP also provides a theoretical foundation for evaluating cardiovascular conditions with fewer assumptions than other methods, and can be further extended into anatomically complex scenarios currently inaccessible by today’s techniques. Moreover, the technique works directly from images with minimal user input required. For clinical use, the validation study revealed that *ν*WERP can capture important clinical biomarkers such as peak relative pressure drop at high accuracy, as well as the transient behaviour of the flow.

Relative pressure estimations are part of routine clinical practice when assessing scenarios such as the degree of valvular stenosis or the severity of aortic coarctation. However, current clinical techniques have shown significant limitations when applied to complex hemodynamic conditions. Doppler ultrasound systematically overestimates valvular pressure drops when severe stenosis skews the flow profile^16^. Conversely, invasive catheterisation of CoA patients is restricted to severe symptomatic cases due to the inherent risks of the procedure. Refined techniques such as the unsteady Bernoulli, iterative methods, PPE or WERP have fundamental limitations illustrated in our results and reported previously^29^. Comparatively, *ν*WERP shows improved versatility, accuracy and robustness *in-silico* and *in-vivo*, extending the feasibility of an accurate and non-invasive approach into challenging anatomies such as the narrow subclavian and renal arteries of the *in-silico* evaluations; situations where implementation of previously proposed techniques would violate fundamental assumptions inherent to these methods. *ν*WERP sensitivity also proved to be stable under realistic image noise scenarios (Figs 3 and 4).

*In-silico* evaluation also enabled the systematic analysis of the required spatiotemporal image resolution of *ν*WERP. As noted previously, performance deteriorates slightly with small lumen diameters where spatial resolution can limit the capacity for images to represent vascular morphology. Moreover, limitations on data such as noise and boundary wall effects will affect *ν*WERP performance. Optimal acquisition settings will also depend on the flow physics: when assessing domains of low flow with significant anatomical constriction, higher spatial resolution is needed to capture morphology and flow. In contrast, higher temporal resolution may be required if quick transient cardiac events occur. The versatility of *ν*WERP allows for adaption to specific cases, providing advantages compared to other routine methods. This approach also allows for more complete hemodynamic analysis to be performed for improved disease risk stratification.

Our comparisons to invasive catheterisation revealed that *ν*WERP can capture the temporal shifts of *in-vivo* aortic relative pressure. Results are similar to the *in-silico* evaluations, with the minor error increases potentially spurring from data acquisition and analysis (image segmentation or co-registration between imaging and catheterisation). Regardless, the clinically important biomarker peak pressure drop is still captured with high accuracy and comparing to other proposed methods, *ν*WERP shows a defined improved performance.

The *in-vivo* cohort represents a particularly challenging study case, since the aortic anatomy of the adolescent subjects present narrow vascular segments (on average ~4 voxels through the diameter). Additionally, the Fontan procedure performed in 3 of the 5 subjects typically result in abnormal ascending aortic vessel morphology and flow profiles^39^, complicating the assessed hemodynamics. Based on this, the *ν*WERP estimates are underlined, with potential applicability spanning over multiple clinical applications. Alternative methods performed poorly in this cohort, largely due to their inherent assumptions. Comparing with situations where simplified Bernoulli estimates are optimised^40^ *ν*WERP show similar or improved levels of accuracy. With that, *ν*WERP represents a viable alternative to current techniques when applied in the same application field, and further enables investigations into more complex, previously inaccessible cardiovascular conditions.

Even though not necessarily restricted to data acquired by 4D flow MRI, the presented *ν*WERP formulation relies on full-field measures of blood flow in the vascular region of interest. An extension of similar work-energy based methods into planar 2D flow acquisitions has been proposed^16^, opening the possibility for similar strategies in the application of *ν*WERP. However, coupling the refinement of *ν*WERP with the rapid development of refined imaging techniques promises the potential of using 3D-based flow methods as standardised diagnostic tools.

To date, the diagnostic usage of relative pressure assessment has been applied for stenotic flow regimes in which the development of turbulent flow plays a role^41^. In severe cases of stenosis, methods incorporating turbulent flow behaviour have shown promising potential to non-invasively assess relative pressure^31,41–43^. In its current form, *ν*WERP does not include analysis of turbulent fluctuations, however the theoretical derivation does allow for the inclusion of a turbulence term originating from the incorporation of incoherent flow variations in the velocity field. Again using the concept of a virtual field, *ν*WERP could thus enable even the assessment of turbulence-driven relative pressures into complex cardiovascular regimes. However, a full derivation, analysis, and method validation remains to be performed, and is beyond the scope of this current work. Instead *ν*WERP is here presented as a method to expand on previous techniques by enabling hemodynamic probing into previously inaccessible vasculatures where the relative pressure drop is a result of combined kinetic, advective, and viscous flow behaviour.

Improvements in medical imaging enable more complete and accurate investigation of hemodynamic biomarkers. Utilising these developments, *ν*WERP represents a novel tool for assessing relative pressure between virtually any connected locations in cardiovascular flow images. The technique demonstrates accuracy and robustness for realistic image quality and resolution over a range of complex cardiovascular conditions *in-silico* and *in-vivo*. Providing improved flexibility and versatility, *ν*WERP constitutes a promising tool opening the possibilities for expansion of hemodynamic biomarkers, both in research scenarios and standardised clinical care.

## Methods

### Theoretical derivation of *ν*WERP

The derivation of *ν*WERP originates from the conservation of mass and momentum for an isothermal viscous Newtonian fluid given by the Navier-Stokes equations, i.e.

1 $$\rho \frac{\partial {\boldsymbol{v}}}{\partial t}+\rho {\boldsymbol{v}}\cdot \nabla {\boldsymbol{v}}-\mu {\nabla }^{2}{\boldsymbol{v}}+\nabla p=0$$

2 $$\nabla \cdot {\boldsymbol{v}}=0$$

where ***v*** is the velocity of the fluid, *p* the fluid pressure, and *μ* and *ρ* are the dynamic viscosity and density, respectively. Derivation of the virtual work-energy equations for such a fluid can be achieved by multiplying (1) by an arbitrary velocity field – or virtual field – ***w*** and integrating over the entire fluid domain Ω, yielding:

3 $${\int }_{{\rm{\Omega }}}\rho \frac{\partial {\boldsymbol{v}}}{\partial t}\cdot {\boldsymbol{w}}d{\rm{\Omega }}+{\int }_{{\rm{\Omega }}}\rho ({\boldsymbol{v}}\cdot \nabla {\boldsymbol{v}})\cdot {\boldsymbol{w}}d{\rm{\Omega }}-{\int }_{{\rm{\Omega }}}\mu {\nabla }^{2}{\boldsymbol{v}}\cdot {\boldsymbol{w}}d{\rm{\Omega }}+{\int }_{{\rm{\Omega }}}\nabla p\cdot {\boldsymbol{w}}d{\rm{\Omega }}=0$$

In this case, equation (3) describes the work-energy within the fluid in the direction of the virtual field ***w***. In the case where ***w*** = ***v***, equation (3) reduces to the classic work-energy equation and provides insight into the actual work and energy held within the fluid at a given time (see Donati *et al*.^32^). Importantly, however, is that the sought relative pressure drop originates from the acquired ***v***, and is not connected to the virtual field, ***w***.

Using integration by parts (converting volume integrals over Ω to surface integrals over the domain boundary Γ with outward normal ***n***), the solenoidal nature of ***v***, and identifying separate virtual energy terms, equation (3) can be concisely written as

4 $$\frac{\partial {K}_{e}}{\partial t}+{A}_{e}-{S}_{e}+{V}_{e}+H(p)=0$$

where

5 $$\frac{\partial {K}_{e}}{\partial t}={\int }_{{\rm{\Omega }}}\rho \frac{\partial {\boldsymbol{v}}}{\partial t}\cdot {\boldsymbol{w}}d{\rm{\Omega }}$$

6 $${A}_{e}={\int }_{{\rm{\Omega }}}\rho ({\boldsymbol{v}}\cdot \nabla {\boldsymbol{v}})\cdot {\boldsymbol{w}}$$

7 $${S}_{e}={\int }_{{\rm{\Gamma }}}(\mu \nabla {\boldsymbol{v}}\cdot {\boldsymbol{n}})\cdot {\boldsymbol{w}}d{\rm{\Gamma }}$$

8 $${V}_{e}={\int }_{{\rm{\Omega }}}\mu \nabla {\boldsymbol{v}}:\nabla {\boldsymbol{w}}d{\rm{\Omega }}$$

9 $$H(p)={\int }_{{\rm{\Gamma }}}p\,{\boldsymbol{w}}\cdot {\boldsymbol{n}}d{\rm{\Gamma }}-{\int }_{{\rm{\Omega }}}p\nabla \cdot {\boldsymbol{w}}\,d{\rm{\Omega }}$$

Here, the intuitive understanding of the different virtual energy terms is most clearly understood in the case where ***w*** = ***v***. In this case, *K*_*e*_ denotes the current kinetic energy held within the fluid, *A*_*e*_ indicates the rate at which kinetic energy enters, exits or grows in the region of interest Ω, *S*_*e*_ indicates the power added or removed from the system due to shear, *V*_*e*_ details the rate of viscous energy dissipation in the fluid and *H*(*p*) defines the hydraulic power.

The calculation in equation (4–9) varies from our previous IMRP estimatior^29^. The key difference resides in equation (6), where we previously applied integration by parts to reduce the numerical derivatives required on imaging data. This, however, lead to a more significant dependence on surface integrals, deteriorating method performance.

To isolate the relative pressure between two boundaries of the region of interest, we seek to assign attributes (or restrictions) on ***w***. Using the desired regions to isolate the pressure drop, the boundary can be split into inlet Γ_*i*_, outlet Γ_*o*_, and wall Γ_*w*_ regions (with Γ = Γ_*i*_ ∪ Γ_*o*_ ∪ Γ_*w*_). Focusing on *H*(*p*), we observe that if the virtual field is a solenoidal field with zero velocity on Γ_*w*_,

10 $$H(p)={\int }_{{{\rm{\Gamma }}}_{i}\cup {\Gamma }_{{o}}}p{\boldsymbol{w}}\cdot {\boldsymbol{n}}d{\rm{\Gamma }}$$

Under these assumptions, applying the Divergence theorem we observe the total inflow, *Q*, given by ***w*** is identical to its outflow, *e.g*.

11 $$0={\int }_{{\rm{\Omega }}}\nabla \cdot {\boldsymbol{w}}d{\rm{\Omega }}={\int }_{{\rm{\Gamma }}}{\boldsymbol{w}}\cdot {\boldsymbol{n}}d{\rm{\Gamma }}={\int }_{{{\rm{\Gamma }}}_{i}\cup {{\rm{\Gamma }}}_{o}}{\boldsymbol{w}}\cdot {\boldsymbol{n}}d{\rm{\Gamma }}$$

Hence,

12 $$H(p)=({p}_{i}-{p}_{o}){\int }_{{{\rm{\Gamma }}}_{i}}{\boldsymbol{w}}\cdot {\boldsymbol{n}}d{\rm{\Gamma }}={\rm{\Delta }}pQ$$

where ${p}_{k}={\int }_{{{\rm{\Gamma }}}_{k}}p{\boldsymbol{w}}\cdot {\boldsymbol{n}}d{\rm{\Gamma }}/{\rm{Q}}$ is the ***w***⋅***n*** weighted mean pressure on the *k*^*th*^ boundary. Further, the conditions on the virtual field reduce *S*_*e*_ to more simplified forms

13 $${S}_{e}={\int }_{{{\rm{\Gamma }}}_{i}\cup {{\rm{\Gamma }}}_{o}}\mu (\nabla {\boldsymbol{v}}\cdot {\boldsymbol{n}})\cdot {\boldsymbol{w}}d{\rm{\Gamma }}$$

In this work, we sought to minimise the added power due to *S*_*e*_. By ensuring that the virtual field acts in the surface normal direction, we focus on the added power due to flow gradients along the length of the artery of interest. As these variations are small, the added power becomes negligible.

Combining equations (5, 6, 8, 12–13), we can express the relative pressure drop as

14 $${\rm{\Delta }}p=-\,\frac{1}{Q}(\frac{\partial }{\partial t}{K}_{e}+{A}_{e}+{V}_{e})$$

meaning that the relative pressure, Δ*p*, is now expressed as a function of the virtual work-energy performed by ***w***.

Apart from acquiring the velocity field ***v***, all that remains for deriving Δ*p* is then to find a virtual field, ***w***, abiding to the assumptions outlined above. In theory, any virtual field fulfilling these criteria can be selected and, in the absence of noise and sampling errors, will result in the same relative pressure estimate. In this study, we elect to construct ***w*** by solving Stokes problem, which ensures the existence of a unique solution satisfying the physical constraints outlined. Specifically, we solve the boundary value problem

15 $${\nabla }^{2}{\boldsymbol{w}}+\nabla \lambda =0$$

16 $$\nabla \cdot {\boldsymbol{w}}=0$$

17 $${\boldsymbol{w}}=\{\begin{array}{ll}-n,\, & {{\rm{\Gamma }}}_{i}\\ 0, & \,{{\rm{\Gamma }}}_{w}\end{array}$$

where *λ* is the virtual pressure field (Lagrange multiplier) corresponding to the virtual field ***w*** used to enforce equation (15), and *n* is the normal vector on the inlet plane Γ_*i*_. Information on the practical implementation of the above is given below under *Data pre-processing and practical implementation of ν*W*ERP*.

### Data pre-processing and practical implementation of *ν*WERP

Below we outline the pre-processing steps and implementation of *ν*WERP for both in silico and *in-vivo* flow fields.

#### Flow field corrections

Using 4D flow MRI (as in the current study), eddy currents, field inhomogeneities, and potential regions of aliasing have to be corrected using customised pre-processing tools. For this specific study, 4D flow fields were reconstructed and corrected using GTFlow (GyroTools LLC).

#### Segmentation of fluid domain

The vascular region of interest required segmentation, separating it from the surrounding tissues and airways. A range of segmentation techniques exist in literature^44^. For this study, segmentation was performed by the following protocol:

1. An initial binary mask was created by collapsing the temporal dimension of the 4D flow velocity magnitude, leaving a spatial image of the average velocity magnitude of each pixel. A moving threshold was applied, removing voxels with velocity magnitude <25–40% of the image velocity encoding (or VENC), with the exact threshold defined by the user.
2. Isolated regions consisting of 10 voxels or less were removed, and remaining open enclosed regions within the remaining binary masks were filled.
3. Distinction of the vessel of interest was achieved by manual selection, separating the vascular segment from other disconnected regions. The vascular segment was isolated using a region growing approach within the binary mask generated from the previous step.
4. The remaining segmented velocity field was evaluated to identify potential erroneous voxels based on examination of:
  - The temporal derivative of the velocity field. For this, temporal derivatives were evaluated throughout the entire mask, and ordered with respect to magnitude creating a velocity-rate histogram. Border voxels where subsequently removed from the mask when the velocity temporal derivative magnitudes were outside two standard deviations of the distribution mean.
  - The predominant flow direction in a given voxel. For this, the mean flow direction in a given voxel at peak systole was compared to the average flow direction in the neighbouring voxel area (within a spherical area of radius <5 voxels). If a voxel’s mean flow direction deviated more than 120° from that of the neighbourhood, the voxel was deemed erroneous and removed. Subsequently, a second check was performed by evaluating the remaining masked voxels against a solved Stokes flow from inlet to outlet. Again, if a singular voxel’s mean flow direction deviated more than 120° from that of the Stokes flow within a <5 voxel radius, it was deemed erroneous and removed.

With this, a final binary mask was created, isolating the fluid domain of interest from static tissue or spurious noise in the acquired flow field.

#### Selection of inlet and outlet planes

Inlet and outlet planes within the binary mask were chosen by manually selecting an input/output point on the surface of the binary mask. The centre-line of the vessel was then automatically identified in the vicinity of the selected points. Using this, a spatial plane was then automatically identified, cutting the vascular cross-section perpendicular to the centre-line direction of the vessel, and including the selected input/output point on the mask surface.

#### Labelling the fluid domain

To facilitate for the definition of boundary conditions within the computation of the virtual field (see *e) Computation of virtual fields* below) a labelling of the vascular domain is performed, making use of the aforementioned domain, inlet, and outlet mask, respectively. Specifically, region growing technique was applied to create a mask nested within the original segmentation and bridging between the inlet and outlet planes. With this, labels are assigned to individual voxels to separate: interior voxels (entirely within the fluid), exterior voxels (entirely outside the fluid), inlet/outlet voxels, and wall voxels (separating interior and exterior domain). With this, appropriate boundary conditions can be set within *e) Computation of virtual fields*.

#### Computation of virtual fields

As described in *Theoretical derivation of ν*W*ERP*, the virtual velocity field ***w*** is chosen to satisfy a Stokes problem (given by equations (15–17)). In this work, the problem is set up and solved numerically using the Finite Difference Method (FDM) (see Supplement Material B). For this, an initial base-sampling of nodal points is generated at each voxel inside the segmented acquired velocity field. To improve on the accuracy of the solved virtual field, a subsampling of the data is performed, subdividing base-voxels by a chosen integer value, making a single image voxel consist of several uniformly distributed nodal points (within this work, all virtual fields were subsampled to an image resolution of 0.5 mm^3^). Note that this subsampling only effects the resolution of ***w*** and all analysis of ***v*** is kept in the given image resolution of the performed acquisition.

With nodal points defined, boundary conditions of the FDM were set such that nodal points on the binary inlet and wall masks were set based on the velocity constraint in equation (17). The virtual field ***w*** can be computed by solving a linear system of equations generated by the FDM. Further details of the method and solver can be found in Supplementary Material. An overview of the pre-processing from segmentation to computed virtual field is also provided in Supplementary Fig. 1.

#### Noise filtering and data smoothing

To reduce the effects of spurious noise in the acquired flow field, spatial noise filtering is applied to the data prior to *ν*WERP estimation. Specifically, a *k*-order 3D polynomial fitting is used over local kernels to approximate a noise-free signal (see Supplementary Fig. 2). The technique applied uses a standard Savitzky-Golay filtering^45^ commonly applied in image processing. For a given flow field, *optimal* interpolation order and kernel size was determined by evaluating the filtering on a 1D sinusoidal signal, with discretisation and maximum spatial gradient identical to that of the input data. Truncated Gaussian noise resembling the SNR of the input data is added to the 1D signal, and noise filtering using polynomial order from 0–3, and kernel sizes from 1^3^ to 9^3^ were evaluated. The combination of polynomial order and kernel size minimising the error to the known 1D sinusoidal function is then chosen as the optimal filter parameters for the given flow field. This choice of optimal filtering parameters was performed once for an entire 4D dataset.

#### νWERP discretisation

The quantification of relative pressure from equation (14) for a spatiotemporally discrete flow field is defined as:

18 $${\rm{\Delta }}{p}_{t+\frac{1}{2}}=-\,\frac{1}{Q({\boldsymbol{w}})}(\frac{\partial }{\partial t}{K}_{e}({{\boldsymbol{v}}}_{t+\frac{1}{2}},{\boldsymbol{w}})+{A}_{e}({{\boldsymbol{v}}}_{t+\frac{1}{2}},{\boldsymbol{w}})+{V}_{e}({{\boldsymbol{v}}}_{t+\frac{1}{2}},{\boldsymbol{w}}))$$

with

19 $${{\boldsymbol{v}}}_{t+\frac{1}{2}}=\frac{1}{2}({{\boldsymbol{v}}}_{t+1}+{{\boldsymbol{v}}}_{t})$$

being the flow and virtual field at a time half-way between *t* and *t* + 1. Note that computations are performed at the midpoint between time steps to improve temporal accuracy.

#### Surface and volume integral estimation

Energy components of *ν*WERP in equation (18) are acquired by integration over the voxelised version of Ω, denoted Ω_*ROI*_. Similarly, surface integrals are evaluated on discretised inlet and outlet planes, Γ_*I*,*ROI*_ and Γ_*O*,*ROI*_, with corresponding normal vectors ***N***. Discretised terms can be computed from ***v*** and ***w*** at voxel (*i*, *j*, *k*) by:

20 $${K}_{e}({\boldsymbol{v}},{\boldsymbol{w}})=\rho dV\sum _{(i,j,k)\,{\epsilon }\,{{\rm{\Omega }}}_{ROI}}({\boldsymbol{v}}(i,j,k)\cdot {\boldsymbol{w}}(i,j,k))$$

21 $$\begin{array}{c}{A}_{e}({\boldsymbol{v}},{\boldsymbol{w}})\,=\,\rho dS\sum _{(i,j)\,{\epsilon }\,{{\rm{\Gamma }}}_{I,ROI}\,\cup \,{{\rm{\Gamma }}}_{O,ROI}}(({\boldsymbol{v}}(i,j)\cdot {\boldsymbol{w}}(i,j))({\boldsymbol{v}}(i,j)\cdot {\boldsymbol{N}}(i,j)))\\ \,\,\,\,\,-\,\rho dV\,\sum _{(i,j,k)\,{\epsilon }\,{{\rm{\Omega }}}_{ROI}}({\boldsymbol{v}}(i,j,k)\cdot G({\boldsymbol{w}})(i,j,k)\cdot {\boldsymbol{v}}(i,j,k))\end{array}$$

22 $${V}_{e}({\boldsymbol{v}},{\boldsymbol{w}})=\mu dV\sum _{(i,j,k)\,{\epsilon }\,{{\rm{\Omega }}}_{ROI}}(G({\boldsymbol{v}})(i,j,k):G({\boldsymbol{w}})(i,j,k)$$

23 $$Q({\boldsymbol{w}})=dS\sum _{(i,j)\,{\epsilon }\,{{\rm{\Gamma }}}_{O,ROI}}({\boldsymbol{w}}(i,j)\cdot {\boldsymbol{N}}(i,j))$$

where $dS=\prod _{i=1}^{2}{\rm{\Delta }}{x}_{i}$ and $dV=\prod _{i=1}^{3}{\rm{\Delta }}{x}_{i}$ are the pixel surface and voxel volume, respectively, based on the voxel length Δ*x* in each spatial dimension, respectively.

#### Spatial and temporal gradient estimation

Spatial derivatives were estimated using a *k*-order 3D polynomial fit with the polynomial order (*k*) and kernel size optimised as discussed above. The gradient of the filtered flow field was subsequently estimated as the gradient of the polynomial fit. For the numerical computation of the spatial gradient of the virtual field, a standard central-difference scheme was applied.

Temporal derivatives were similarly derived using a central-difference approximation. Specifically, estimates were acquired in-between given time sampling points, i.e:

24 $${\frac{\partial {\boldsymbol{v}}}{\partial t}|}_{t+\frac{1}{2}}(i,j,k)=\frac{{{\boldsymbol{v}}}_{t+1}(i,j,k)-{{\boldsymbol{v}}}_{t}(i,j,k)}{{\rm{\Delta }}t}$$

with Δ*t* being the temporal discretisation.

#### Software implementation

*ν*WERP, including all of the processes above, were implemented as a standalone algorithm using MATLAB R2016a (MathWorks, Natick, MA, USA).

### Generation of patient-specific *in-silico* image data

For evaluating *v*WERP, patient-specific *in-silico* data was generated from computational fluid dynamics simulations of a coarcted and an acute type B dissected aorta, respectively. Both models have been presented in detail elsewhere^34,35^, but for completeness a summary of the data generation process is outlined below.

#### Coarcted aorta

With anatomical data originating from 3D contrast enhanced MR angiography, a model of the aortic arch, including connecting carotid bifurcations and details of the pathological coarctation, was created using a combination of SimVascular (simtk.org) and MeshSim (Simmetrix, Clifton Park, NY). Using acquired 2D PC-MRI data, appropriate boundary conditions were set to simulate flow through the coarcted aorta model. Simulations were run in SimVascular using a Coupled-Momentum Method^46^ developed for solving the incompressible Navier-Stokes equations in deformable arteries. For the *ν*WERP analysis, the simulated dataset was sampled onto a uniform grid of 2 mm^3^ voxels, with 22 time slices representing one cardiac cycle.

#### Dissected aorta

Using a combination of thoracic computed tomography angiography (CTA) and 2D PC-MRI, a patient-specific simulation model of an acute type B dissected aorta was segmented, with the model including vascular anatomy spanning from the carotid to the femoral arteries. The model was built using CRIMSON^47^ by defining well-spaced transmural contours along each vessel in the acquired image data. A volume segmentation was produced by lofting the contour sets using non-uniform rational B-splines, and individual vessels were joined to create a full CAD segmentation, including connecting the identified segments^48^. Each lumen was created separately and the final model was visually checked to verify consistency to the image data. 17 connecting tears were introduced between true and false lumen. With that, the incompressible Navier-Stokes equations were solved using a stabilised finite-element formulation. The acquired 2D PC-MRI at the aortic root was used as inlet boundary condition. The distal vascular bed was modelled using 0D Windkessel RCR elements coupled to the 3D domain using a multi-domain method^49^ and attached at all 17 outflow arteries. Parameters for each RCR were tuned to match 2D PC-MRI acquired in a number of positions in the descending aorta. The meshes were created with tetrahedral elements (global element size: 1 mm, local refinements at vascular wall between 0.1–0.05 mm), with iterative mesh adaptation performed at regions experiencing high velocity Hessians. Simulations were run for multiple cycles until a periodic solution was found for both flow and pressure.

For the *ν*WERP analysis, and particularly the spatiotemporal convergence analysis, the simulated dataset was sampled onto a uniform grid of varying spatiotemporal resolution. In silico images were generated for 1, 2, 3, and 4 mm_3_ voxel size, with temporal sampling of between 8 and 64 time slices representing one cardiac cycle.

#### Image noise

For both *in-silico* sets, image noise was applied frame-by-frame using the maximum velocity, *v*_*max*_, of the entire dataset to represent the velocity encoding of a 4D flow MRI acquisition. With such, the relationship between the standard deviation of the velocity field, *σ*_*v*_, and the SNR can be expressed as^50^:

25 $${\sigma }_{v}=\frac{\sqrt{2}{v}_{max}}{\pi \cdot SNR}$$

With that a corresponding *σ*_*v*_ could be derived for the low- and high-noise configurations, respectively. Knowing this, noise was applied distributed over all voxels of the in silico flow images, following a truncated Gaussian distribution^51^, with data truncation applied above two standard deviations to avoid spurious noise outliers.

### *In-vivo* catheter and 4D flow MRI data acquisition

For the validation of *ν*WERP, data was obtained during a combined cardiac MRI and cardiac catheterisation procedure (XMR). Both cardiac MRI imaging and invasive pressure assessments were performed under the same general anaesthesia within a timeframe of 30 minutes to one hour. In all cases heart rate and blood pressures were constantly monitored and remained stable throughout the procedure. Invasive pressures were obtained using pullback procedures from the ventricle into the ascending aorta, proximal descending aorta, diaphragmatic aorta and the femoral artery in steps using a fluid filled catheter (4 F GL braided catheter, Terumo Medical Corp., Somerset, NJ). 10 second pressure readings were obtained at each site, with multiple beats acquired throughout the respiratory cycle. Using the simultaneously acquired ECG signals, pressure measurements at different anatomical positions could be co-registered (performed by forcing signal overlap at corresponding peak R-wave positions). At each catheter position, multiple curves were acquired throughout the respiratory cycle. Instantaneous relative pressure differences were then computed by subtracting pressures in the proximal measurement point with pressures in the more distal measurement point(s). This process was repeated to generate a family of difference curves from which mean and standard deviations were extracted to represent the entire set of catheter measurements. The position of the catheter was visually confirmed by fluoroscopy imaging, used to guide the positioning of the catheter during *in-vivo* measurements, and manually identified in the corresponding segmented flow field from the 4D flow MRI.

The 4D flow MRI data was acquired using a Philips Achieva system (1.5 T, average spatial resolution: 2.1 mm^3^, temporal resolution: ~31 ms), with complete flow fields reconstructed using GTFlow (GyroTools Ltd, Zurich, Switzerland), in which eddy current, field inhomogeneities, and potential aliasing were corrected for.

For the temporal alignment of 4D flow MRI data and cardiac catheterisation, this was achieved by forced overlap at peak systole, with potential cardiac phase extensions unwrapped to generate data within one given cardiac cycle only. Apart from this, the temporal sampling rate of the two measurements were kept separate, given by the used measurement equipment, respectively.

Note that all included patients underwent a clinically indicated XMR for comprehensive assessment of cardiac and vascular function for unexplained exercise intolerance in the face of a complex congenital heart disease. The subject characteristics of the 6 patients included in this study are summarised in Supplementary Table 1. As stated in the Results section, all patients participated under informed consent, with study approval granted by the Regional Ethics Committee, South East London, United Kingdom (REC, 10/H0802/65). Additionally, all methods and experiments were performed in accordance with relevant guidelines and regulations.

### Data analysis

The mean error, *ε*_*Δp*_, for the temporal *ν*WERP estimations comparing either against available *in-silico* or *in-vivo* data is defined as:

26 $${\varepsilon }_{{\rm{\Delta }}p}=(\frac{\sqrt{{\sum }_{n=1}^{N}\,{({{\rm{\Delta }}{p}_{W}|}_{n}-{{\rm{\Delta }}p|}_{n})}^{2}}}{\sqrt{{\sum }_{n=1}^{N}\,{{{\rm{\Delta }}p|}_{n}}^{2}}})\cdot 100$$

where Δ*p*_*W*_|_*n*_ is the *ν*WERP estimate at discrete time step *t*_*n*_ and Δ*p*|_*n*_ is the corresponding true data closest to the *ν*WERP estimate within *t*_*n*−1_ < *t* < *t*_*n*+1_, with closeness evaluated by minimising the Euclidean distance between the temporal curve of the true and *ν*WERP pressure drop, respectively. Note that for temporal estimations over an entire cardiac cycle, *T*, the above is evaluated at discrete temporal acquisitions *t*_1_ to *t*_*N*_ where N is the number of temporal sample points.

The root mean square error, *ε*_*rms*_, is calculated by:

27 $${\varepsilon }_{rms}=\sqrt{\frac{{\sum }_{n=1}^{N}\,{({\rm{\Delta }}{p}_{vWERP}({t}_{n})-{\rm{\Delta }}p({t}_{n}))}^{2}}{N}}\cdot 100$$

For the derivation of the error at the pressure peak, ${\varepsilon }_{{\rm{\Delta }}{p}_{max}}$, this was defined as:

28 $${\varepsilon }_{{\rm{\Delta }}{p}_{max}}=|\frac{{\rm{\Delta }}{p}_{W,max}-{\rm{\Delta }}{p}_{max}}{{\rm{\Delta }}{p}_{max}}|\cdot 100$$

Using the maximum relative pressure peak, Δ*p*_*max*_, and corresponding relative pressure peak estimated by *ν*WERP, Δ*p*_*W*,*max*_, as input. Again the *ν*WERP estimate was identified within *t*_*n*−1_ < *t* < *t*_*n*+1_, with closeness evaluated by minimising the Euclidean distance between the temporal curve of the true and *ν*WERP pressure drop, respectively.

### Alternative methods for non-invasive relative pressure estimation

In this work, *ν*WERP performance is compared to alternative methods proposed for non-invasive relative pressure estimation. Details for these methods are given in other references^24,25,32,37,52,53^. Below is a short summary of the used methods.

#### Bernoulli

The Bernoulli equation^52,53^, commonly used clinically as part of recommended guidelines^1^, assumes that the relative pressure, Δ*p*, is governed solely by the advective component of the Navier-Stokes equations. By so, Δ*p* can be derived as:

29 $${\rm{\Delta }}p=\frac{1}{2}\rho ({v}_{1}^{2}-{v}_{2}^{2})$$

With *v*_2_ and *v*_1_ the velocity at a single point of the inlet and outlet plane, respectively, and *ρ* the fluid viscosity.

#### Unsteady Bernoulli

Extending from the above given Bernoulli equation, the unsteady Bernoulli^37^ incorporates contributions due to inertial accelerations, with the pressure drop defined as

30 $${\rm{\Delta }}p=\frac{1}{2}\rho ({v}_{1}^{2}-{v}_{2}^{2})-\rho {\int }_{S}\frac{\partial v}{\partial t}\,ds$$

The influence of the inertial acceleration is then added by evaluating the temporal derivatives of the flow along the trace, *S*, of an infinitesimal particle seeded into the flow. For simplicity, this streamline is often assumed to follow the centreline of the investigated vessel.

#### Work Energy Relative Pressure (WERP)

The Work Energy Relative Pressure (WERP)^32^ is the predecessor to the proposed *ν*WERP. The theory of WERP is identical to that provided in *Theoretical derivation of ν*W*ERP*. However, a key differences lies in that no virtual field is introduced, but rather the work-energy of the acquired flow field ***v*** is evaluated. By so, Δ*p* is defined as:

31 $${\rm{\Delta }}p=-\,\frac{1}{Q}(\frac{\partial }{\partial t}{K}_{e}+{A}_{e}+{V}_{e})$$

Compared to *ν*WERP, all energy terms above now relate to ***v***. Identically, *Q* is the flow of ***v*** over the defined inlet/outlet planes, with divergent behaviour seen when *Q* → 0.

## Supplementary information

Supplementary material

## Data Availability

All data generated or analysed during this study are included in this published article (and its Supplementary Information files).

## References

[1] Baumgartner H (2017). ESC/EACTS Guidelines for the management of valvular heart disease. European Heart Journal.

[2] Bernard J, Barry J, Joseph A (2011). ACCF/AHA Guideline for the Diagnosis and Treatment of Hypertrophic Cardiomyopathy. JACC.

[3] Vahanian A (2007). Guidelines on the management of valvular heart disease: The Task Force on the Management of Valvular Heart Disease of the European Society of Cardiology. European heart journal.

[4] De Bruyne B (2006). Assessment of renal artery stenosis severity by pressure gradient measurements. Journal of the American College of Cardiology.

[5] Tan J-L, Babu-Narayan SV, Henein MY, Mullen M, Li W (2005). Doppler echocardiographic profile and indexes in the evaluation of aortic coarctation in patients before and after stenting. Journal of the American College of Cardiology.

[6] Jenkins NP, Ward C (1999). Coarctation of the aorta: natural history and outcome after surgical treatment. QJM: An International Journal of Medicine.

[7] Nishimura, R. A. *et al*. AHA/ACC Guideline for the management of patients with valvular heart disease: executive summary. *Circulation*, CIR. 0000000000000029 (2014). 10.1161/CIR.0000000000000029 24589852

[8] Feldman T (2006). Assessment of the transvalvular pressure gradient in aortic stenosis. J. Invasive Cardiol.

[9] Wyman RM (1988). Current complications of diagnostic and therapeutic cardiac catheterization. Journal of the American College of Cardiology.

[10] Vitiello R, McCrindle BW, Nykanen D, Freedom RM, Benson LN (1998). Complications associated with pediatric cardiac catheterization. Journal of the American College of Cardiology.

[11] Omran H (2003). Silent and apparent cerebral embolism after retrograde catheterisation of the aortic valve in valvular stenosis: a prospective, randomised study. The Lancet.

[12] Yock PG, Popp RL (1984). Noninvasive estimation of right ventricular systolic pressure by Doppler ultrasound in patients with tricuspid regurgitation. Circulation.

[13] Panza JA, Petrone RK, Fananapazir L, Maron BJ (1992). Utility of continuous wave Doppler echocardiography in the noninvasive assessment of left ventricular outflow tract pressure gradient in patients with hypertrophic cardiomyopathy. Journal of the American College of Cardiology.

[14] Baumgartner H, Stefenelli T, Niederberger J, Schima H, Maurer G (1999). “Overestimation” of catheter gradients by Doppler ultrasound in patients with aortic stenosis: a predictable manifestation of pressure recovery. Journal of the American College of Cardiology.

[15] Garcia D, Dumesnil JG, Durand L-G, Kadem L, Pibarot P (2003). Discrepancies between catheter and Doppler estimates of valve effective orifice area can be predicted from the pressure recovery phenomenon: practical implications with regard to quantification of aortic stenosis severity. Journal of the American College of Cardiology.

[16] Donati F (2017). Beyond Bernoulli. Circulation: Cardiovascular Imaging.

[17] Gill RW (1985). Measurement of blood flow by ultrasound: accuracy and sources of error. Ultrasound in medicine & biology.

[18] Holen J, Simonsen S (1979). Determination of pressure gradient in mitral stenosis with Doppler echocardiography. Heart.

[19] Stankovic Z, Allen BD, Garcia J, Jarvis KB, Markl M (2014). 4D flow imaging with MRI. Cardiovascular diagnosis and therapy.

[20] Provost J (2014). 3D ultrafast ultrasound imaging *in vivo*. Physics in medicine and biology.

[21] Ebbers T, Farnebäck G (2009). Improving computation of cardiovascular relative pressure fields from velocity MRI. Journal of Magnetic Resonance Imaging.

[22] Meier, S. *et al*. In Computing in Cardiology, 2010 903–906 (IEEE, 2010).

[23] Yang GZ, Kilner PJ, Wood NB, Underwood SR, Firmin DN (1996). Computation of flow pressure fields from magnetic resonance velocity mapping. Magnetic resonance in medicine.

[24] Krittian SBS (2012). A finite-element approach to the direct computation of relative cardiovascular pressure from time-resolved MR velocity data. Medical Image Analysis.

[25] Bock J (2011). *In vivo* noninvasive 4D pressure difference mapping in the human aorta: phantom comparison and application in healthy volunteers and patients. Magnetic resonance in medicine.

[26] Lamata P (2014). Aortic relative pressure components derived from four‐dimensional flow cardiovascular magnetic resonance. Magnetic resonance in medicine.

[27] Riesenkampff E (2014). Pressure Fields by Flow-Sensitive, 4D, Velocity-Encoded CMR in Patients With Aortic Coarctation. JACC: Cardiovascular Imaging.

[28] Donati, F., Nordsletten, D. A., Smith, N. P. & Lamata, P. In 2014 36th Annual International Conference of the IEEE Engineering in Medicine and Biology Society 5097–5100 (IEEE, 2014). 10.1109/EMBC.2014.6944771 PMC6217920 25571139

[29] Bertoglio, C., Núņez, R., Galarce, F., Nordsletten, D. & Osses, A. Relative pressure estimation from velocity measurements in blood flows: state-of-the-art and new approaches (2016). 10.1002/cnm.2925 28884520

[30] Švihlová H, Hron J, Málek J, Rajagopal K, Rajagopal K (2016). Determination of pressure data from velocity data with a view toward its application in cardiovascular mechanics. Part 1. Theoretical considerations. International Journal of Engineering Science.

[31] Ha, H. *et al*. Estimating the irreversible pressure drop across a stenosis by quantifying turbulence production using 4D Flow MRI. *Scientific Reports* 7 (2017). 10.1038/srep46618 PMC5397859 28425452

[32] Donati F, Figueroa CA, Smith NP, Lamata P, Nordsletten DA (2015). Non-invasive pressure difference estimation from PC-MRI using the work-energy equation. Medical image analysis.

[33] Cenic A, Nabavi DG, Craen RA, Gelb AW, Lee T-YA (2000). CT method to measure hemodynamics in brain tumors: validation and application of cerebral blood flow maps. American Journal of Neuroradiology.

[34] Sotelo JA (2015). Pressure gradient prediction in aortic coarctation using a computational-fluid-dynamics model: validation against invasive pressure catheterization at rest and pharmacological stress. Journal of Cardiovascular Magnetic Resonance.

[35] Dillon-Murphy D, Noorani A, Nordsletten D, Figueroa CA (2016). Multi-modality image-based computational analysis of haemodynamics in aortic dissection. Biomechanics and modeling in mechanobiology.

[36] Hagan PG (2000). The international registry of acute aortic dissection (irad): New insights into an old disease. JAMA.

[37] Firstenberg MS (2000). Noninvasive estimation of transmitral pressure drop across the normal mitral valve in humans: importance of convective and inertial forces during left ventricular filling. Journal of the American College of Cardiology.

[38] de Vecchi A (2014). Catheter-induced errors in pressure measurements in vessels: an *in-vitro* and numerical study. IEEE Transactions on Biomedical Engineering.

[39] Itatani K (2012). Influence of surgical arch reconstruction methods on single ventricle workload in the Norwood procedure. The Journal of thoracic and cardiovascular surgery.

[40] Sasson Z, Yock PG, Hatle LK, Alderman EL, Popp RL (1988). Doppler echocardiographic determination of the pressure gradient in hypertrophic cardiomyopathy. Journal of the American College of Cardiology.

[41] Dyverfeldt P, Hope MD, Tseng EE, Saloner D (2013). Magnetic resonance measurement of turbulent kinetic energy for the estimation of irreversible pressure loss in aortic stenosis. JACC: Cardiovascular Imaging.

[42] Ha, H., Kvitting, J. P. E., Dyverfeldt, P. & Ebbers, T. Validation of pressure drop assessment using 4D flow MRI‐based turbulence production in various shapes of aortic stenoses. *Magnetic resonance in medicine* (2018). 10.1002/mrm.27437 30252155

[43] Gülan U, Binter C, Kozerke S, Holzner M (2017). Shear-scaling-based approach for irreversible energy loss estimation in stenotic aortic flow–an *in vitro* study. Journal of biomechanics.

[44] Byrne N, Velasco Forte M, Tandon A, Valverde I, Hussain T (2016). A systematic review of image segmentation methodology, used in the additive manufacture of patient-specific 3D printed models of the cardiovascular system. JRSM Cardiovascular Disease.

[45] Savitzky A, Golay MJ (1964). Smoothing and differentiation of data by simplified least squares procedures. Analytical chemistry.

[46] Figueroa CA, Vignon-Clementel IE, Jansen KE, Hughes TJR, Taylor CA (2006). A coupled momentum method for modeling blood flow in three-dimensional deformable arteries. Computer Methods in Applied Mechanics and Engineering.

[47] CRIMSON The software for cardiovascular modelling and simulation n.d, www.crimson.software.

[48] Wang KC, Dutton RW, Taylor CA (1999). Improving geometric model construction for blood flow modeling. IEEE Engineering in Medicine and Biology Magazine.

[49] Vignon-Clementel IE, Figueroa CA, Jansen KE, Taylor CA (2006). Outflow boundary conditions for three-dimensional finite element modeling of blood flow and pressure in arteries. Computer methods in applied mechanics and engineering.

[50] Andersen A, Kirsch J (1996). Analysis of noise in phase contrast MR imaging. Medical Physics.

[51] Robert CP (1995). Simulation of truncated normal variables. Statistics and computing.

[52] Rijsterborgh H, Roelandt J (1987). Doppler assessment of aortic stenosis: Bernoulli revisited. Ultrasound in medicine & biology.

[53] Hatle L, Brubakk A, Tromsdal A, Angelsen B (1978). Noninvasive assessment of pressure drop in mitral stenosis by Doppler ultrasound. Heart.

