# Supplementary material for: Estimation of Cardiovascular Relative Pressure Using Virtual Work-Energy
Source: Sci Rep. 2019 Feb 4;9:1375. doi: 10.1038/s41598-018-37714-0 (PMC6362021; doi:10.1038/s41598-018-37714-0)
Supplement: Supplementary file 1 — Supplementary material [file 41598_2018_37714_MOESM1_ESM.pdf]

Supplementary material

Estimation of Cardiovascular Relative Pressure Using Virtual Work-Energy

David Marlevi[1,2], Bram Ruijsink[3,4], Maximilian Balmus[3], Desmond Dillon-Murphy[3], Daniel Fovargue[3], Kuberan Pushparajah[3,4], Cristobal Bertoglio[5], Massimiliano Colarieti-Tosti[1,6], Matilda Larsson[1], Pablo Lamata[3], C. Alberto Figueroa[3,7], Reza Razavi[3,4] and David A. Nordsletten[3,*]

*Corresponding author

[1]Department of Biomedical Engineering and Health Systems,
KTH Royal Institute of Technology, Stockholm, Sweden

[2]Department of Clinical Sciences, Karolinska Institutet, Stockholm, Sweden

[3]Division of Imaging Sciences and Biomedical Engineering, King's College London, St Thomas' Hospital, London, United Kingdom

[4]Department of Congenital Heart Disease, Evelina Children's Hospital, London, United Kingdom

[5]Faculty of Science and Engineering, University of Groningen

[6]Department of Clinical Science, Intervention and Technology (CLINTEC), Karolinska Institutet, Stockholm, Sweden

[7]Departments of Surgery and Biomedical Engineering, University of Michigan, Ann Arbor, USA

Supplementary material

A. Analytical spatiotemporal convergence analysis

The spatiotemporal convergence of *v*WERP was evaluated against analytical solutions where the pressure and flow solution were given. Specifically, the flow through a cylindrical pipe of radius, $R = 15\,mm$, and length, $L = 100\,mm$, was analysed, with two flow configurations: a steady-state, laminar viscous flow, and one unsteady, pulsatile kinetic flow.

For the steady-state, laminar viscous flow, the analytical solution was provided by the Hagen-Poiseuille equation where the axial velocity, v_z, is defined as:

$$v_z(r) = \frac{1}{4\mu}\frac{dp}{dz}(R^2 - r^2) \tag{A}$$

with μ denoting the dynamic viscosity, dp/dz the pressure gradient, and r denoting the radial coordinate. For the analytical tests, dynamic viscosity $\mu = 0.004$ Pa·s and pressure gradient $dp/dz = 71.1$ Pa/m^2, was chosen. The flow was discretised on voxel grids of 1,2,3 or 4 mm^3 and truncated Gaussian noise was added to generate sets of SNR = 10/30/∞.

For the pulsatile kinetic flow, a Womersley flow was evaluated, where v_z is given by

$$v_z(r,t) = \frac{1}{4\mu}\frac{dp}{dz}\bigg|_0 (R^2 - r^2) + Re\left\{\sum_{n=1}^{N} i\frac{1}{\rho\omega_n}\frac{dp}{dz}\bigg|_n \left[1 - \frac{J_0\left(\alpha_n i^{3/2}\frac{r}{R}\right)}{J_0(\alpha_n i^{3/2})}\right] e^{i\omega_n t}\right\} \tag{B}$$

$$\alpha_n = R\sqrt{\frac{\omega_n \rho}{\mu}}, \quad \omega_n = n\omega \tag{C}$$

with $\left.\frac{dp}{dz}\right|_n$ representing the pressure gradient at angular frequency ω_n, with a corresponding Womersley number α_n, and J_0 being the Bessel function of zero-th order. To mimic a realistic pulse shape, an aortic mean inflow curve from a PC-MRI measurement of a healthy aorta was decomposed into characteristic Fourier frequencies, and with ω_n identified, (B) could be solved for the entire analysed pipe. As previously, the flow was discretised on voxel grids of 1, 2, 3 or 4 mm^3 with temporal sampling performed at 8, 16, 32, and 64 time points over the analysed cycle. Truncated Gaussian noise was added for sets of SNR = 10/30 /∞.

The results are summarised in Supplementary Table 2. For the steady-state, laminar viscous flow, accuracy deteriorates with added noise. νWERP estimates show average errors of below ~47% in the low-noise scenario at lowest spatial sampling, with results deteriorating further in the high-noise scenario. Importantly, the pressure drop of the analysed viscous flow will be governed completely by the viscous energy component of νWERP (see Online Methods). With that, the accuracy of the pressure drop will be directly dependent on the accuracy with which the spatial derivatives are captured – a feature sensitive to spurious acquisition noise and spatial resolution. While this highlights a demand on spatial resolution and SNR, the instances of purely viscous flows in the cardiovascular system are limited to vascular compartments that are largely inaccessible to current full-field measurement techniques.

For the pulsatile kinetic flow, the results seem less dependent on added image noise. Instead, spatiotemporal sampling has a clear impact on the evaluated estimates. In the noise-free configuration, a two-fold increase in spatial sampling results in an average

10% increase in the relative pressure accuracy, whereas a two-fold increase in temporal sampling results in an average 50% increase in accuracy. For both high- and low-noise configurations, similar behavior is seen with a 9% increase with spatial resampling, and 49% for similar temporal resampling. In this case, the kinetic term in the νWERP formulation dominates, with advective terms appearing only in the presence of noise, and viscous terms orders of magnitude smaller than the stronger kinetic terms. Hence, contrasting the purely viscous test, this principally highlights the influence of temporal sampling. In general, the kinetic evaluation shows that at clinically used spatiotemporal sampling rates (2mm^3, 32 temporal sample points), errors are at or below 10% for all of the analysed noise configurations. The simplicity of the flow field, in this case, however, limits its extension to the more realistic scenarios observed *in-silico* and *in-vivo*.

B. Numerical construction of finite difference problem, and linear equation solver

In νWERP, the virtual field $\boldsymbol{w}$ is constructed by solving a Stokes problem, as outlined in equations (15-17) in the *Online Methods*. The finite difference method (FDM) is applied to numerically compute the solution, using a staggered grid setup[1]. By this approach, the virtual velocity and virtual pressure nodes are defined at superimposed grids, such that the two are theoretically solved at intersection points.

Assuming a 2D setup, set $\boldsymbol{w} = (w_x, w_y)$. The second order derivatives required for the Laplacian in (16) can then be computed at a discrete node (i, j) by a standard central difference scheme, i.e.:

$$\frac{\partial^2 w_x}{\partial x^2} = \frac{w_x|_{i+1,j} - 2w_x|_{i,j} + w_x|_{i-1,j}}{\Delta x^2} + \frac{w_x|_{i,j-1} - 2w_x|_{i,j} + w_x|_{i,j-1}}{\Delta y^2} \tag{D}$$

and similar for w_y.

The first order derivatives of the virtual pressure field, λ, are computed by a forward difference scheme in each spatial direction, i.e.:

$$\frac{\partial \lambda}{\partial x} = \frac{\lambda|_{i+1,j} - \lambda|_{i,j}}{\Delta x} \tag{E}$$

The divergence condition of equation (16) is approximated by:

$$\nabla \cdot \boldsymbol{w} = \frac{w_x|_{i+1,j} - w_x|_{i,j}}{\Delta x} + \frac{w_y\big|_{i,j+1} - w_y\big|_{i,j}}{\Delta y} \tag{F}$$

Assembling the entire FDM setup, (15-17) can be expressed as a linear algebraic system of equations, i.e.:

$$\begin{pmatrix} A & B^T \\ B & 0 \end{pmatrix} \begin{pmatrix} \boldsymbol{w} \\ \lambda \end{pmatrix} = \begin{pmatrix} R \\ 0 \end{pmatrix} \tag{G}$$

where the sub-block matrices $\mathrm{A} \in \mathbb{R}^{M \times M}$, $\mathrm{B} \in \mathbb{R}^{N \times M}$ and $\mathrm{B}^{\mathrm{T}} \in \mathbb{R}^{M \times N}$ represent the discrete, finite difference forms of the Laplacian, gradient and divergence operators, respectively. Additionally R represents the boundary conditions $\boldsymbol{w}$ (given by equation (17)), and 0 being the corresponding conditions on λ (set to zero in our case, since the pressure solution is redundant for *v*WERP).

In order to reduce the associated memory and computational costs associated with inverting (D) to solve for $\boldsymbol{w}$, we employ an iterative solver strategy based on the

BFBT-preconditioning method[2]. To elucidate the basis of this approach, consider the following LDU block factorisation of the concerned parts of (D):

$$\begin{pmatrix} A & B^T \\ B & 0 \end{pmatrix} = \begin{pmatrix} I_M & 0 \\ BA^{-1} & I_N \end{pmatrix} \begin{pmatrix} A & 0 \\ 0 & -S \end{pmatrix} \begin{pmatrix} I_M & A^{-1}B^T \\ 0 & I_N \end{pmatrix} \tag{H}$$

where $\mathrm{S} = \mathrm{BA}^{-1}\mathrm{B}^{\mathrm{T}}$ is the corresponding Schur complement, and I_k is the identity matrix of rank k. By multiplying the last two terms, we obtain the so-called right-hand preconditioner, P:

$$P = \begin{pmatrix} A & B^T \\ 0 & -S \end{pmatrix} \tag{I}$$

From factorisation, the system resulting from preconditioning has a single-value spectrum. Given this property, the generalised minimal residual algorithm (GMRES) solvers[3] converges in a maximum of two iterations. However, with the exact form requiring an analytical inversion of S, an approximate solution is needed for large-scale system (as is the case for the current *v*WERP application). For such, BFBT approximates the inverse of S by:

$$S^{-1} = (BB^T)^{-1}BAB^T(BB^T)^{-1} \tag{J}$$

Additionally, the fact that A is positive semi-definite and BB^T is positive definite can be exploited and their respective inverses approximated by an algebraic multigrid method (AMG)[4, 5]. In this setup, the grid is constructed with Stuben's direct method[6], while smoothing is achieved via two Gauss-Seidel iterations. The AMG approximation process is repeated for two V-cycles in the case of both blocks.

For the νWERP applications of the present paper, a residual of $1e^{-6}$ is set, solving the equation system and generating the desired $\boldsymbol{w}$.

Numerical tests show that the outlined iterative linear equation solver performs favourably to routinely available direct inversion techniques – in this case the MATLAB (MathWorks, Natick, MA, USA) `mldivide` (commonly known as the backslash operator) when applied on large systems. Specifically, the iterative solver requires 81, 169 and 700 seconds for ranks of 130 000, 219 000 and 590 000, respectively. In comparison, the direct solver requires 187, 565 and 6400 seconds for the same systems, respectively. The above evaluations were all performed on a Macintosh-based machine (macOS Sierra) equipped with an Intel Core i7-7820HQ-processor and a RAM of 16GB of 2133 MHz LPDDR3, using MATLAB R2017a (MathWorks Natick, MA, USA).

References

1. Randall, C. Absorbing boundary condition for the elastic wave equation: Velocity-stress formulation. *Geophysics* **54**, 1141-1152 (1989).
2. Elman, H., Howle, V.E., Shadid, J., Shuttleworth, R. & Tuminaro, R. Block preconditioners based on approximate commutators. *SIAM Journal on Scientific Computing* **27**, 1651-1668 (2006).
3. Saad, Y. & Schultz, M.H. GMRES: A generalized minimal residual algorithm for solving nonsymmetric linear systems. *SIAM Journal on scientific and statistical computing* **7**, 856-869 (1986).
4. Ruge, J.W. & Stüben, K. Algebraic multigrid. *Multigrid methods* **3**, 73-130 (1987).
5. Brandt, A., McCoruick, S. & Huge, J. Algebraic multigrid (AMG) for sparse matrix equati0ns. *Sparsity and its Applications* **257** (1985).
6. Stüben, K. An introduction to algebraic multigrid. *Multigrid*, 413-532 (2001).

Supplementary figures and tables

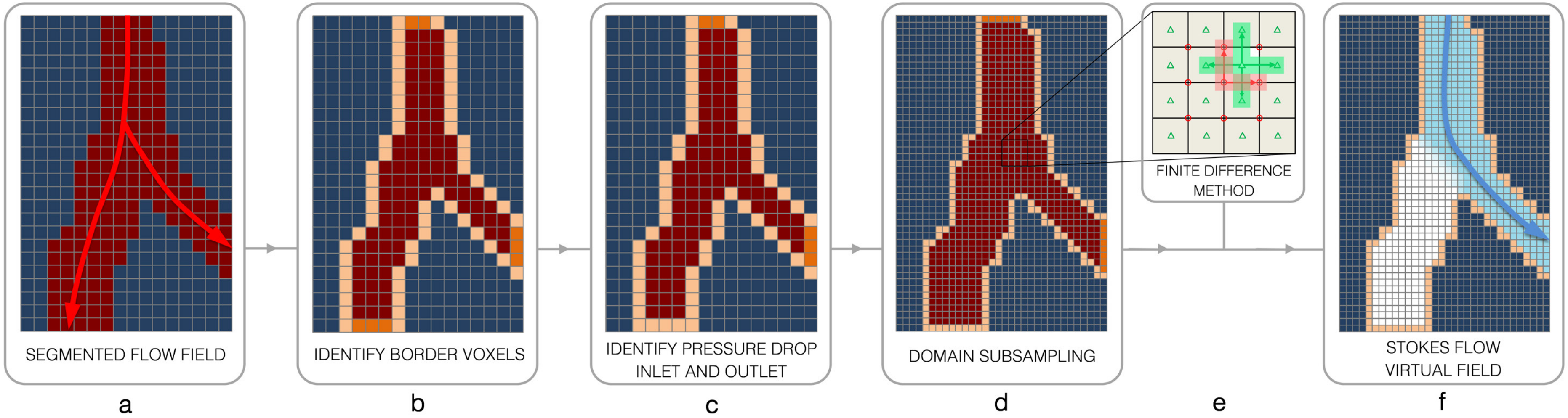

Supplementary figure 1. Method to compute the virtual field used in *v*WERP estimation. **(a)** Segmented flow field, separating pixels of identified flow from surrounding static tissue. **(b)** Identification of border pixels along with inlets and outlets. **(c)** Selection of user inlet and outlet planes from available inlet/outlet boundaries. **(d)** Domain subsampling to improve numerical accuracy of the Stokes flow solution. **(e)** A staggered grid finite difference method is used to approximate the Stokes flow problem and compute virtual field. The velocity nodes are defined by the green triangles, with required derivatives computed by central difference (including neighboring pixels in the shaded green area). The pressure nodes are defined by the red circles, shifted by half a pixel from the velocity nodes. Required derivatives are here computed by a one-sided difference scheme (including neighboring pixels in the shaded red area). **(f)** Final virtual field isolating the flow area over which relative pressures are to be computed.

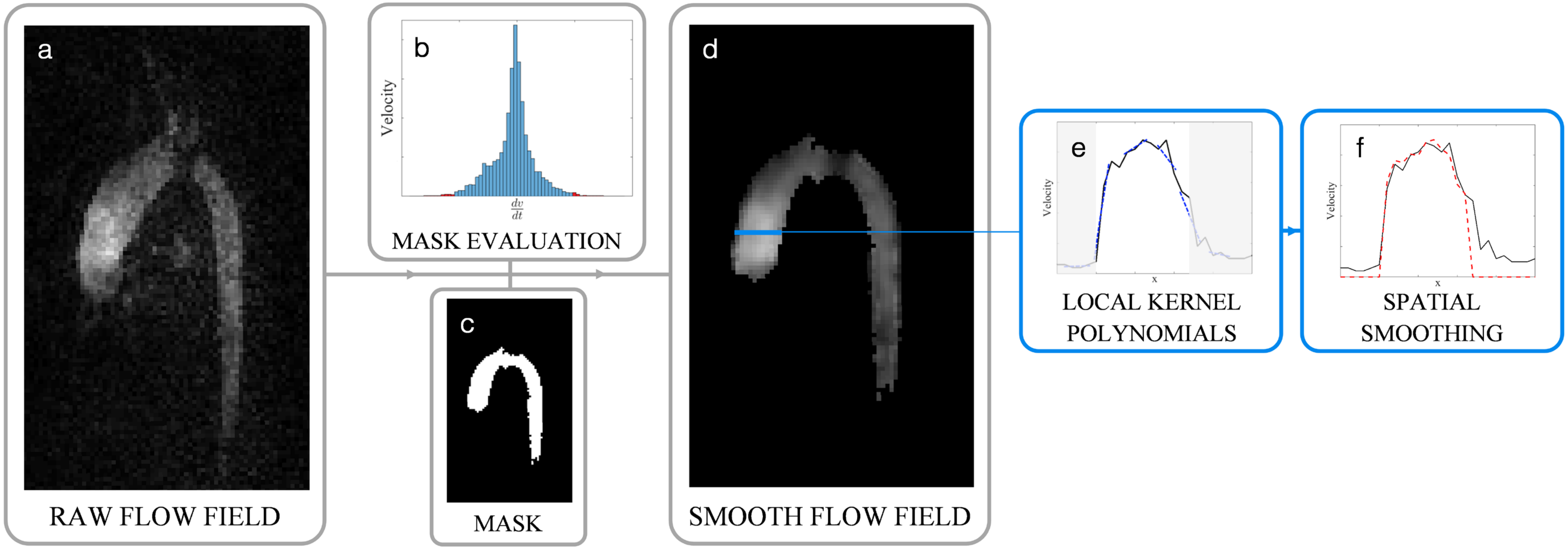

Supplementary figure 2. Segmentation and noise filtering techniques incorporated into the *v*WERP method presented. **(a)** Input raw data flow field. **(b)** Evaluations of temporal derivatives inside the masked flow domain, with identifications of derivatives above 2 standard deviations of the mean. If such a voxel is a voxel bordering the static domain, it is removed from the mask. **(c)** Masking of the fluid domain. **(d)** Smoothed flow field, obtained by a combination of spatial and temporal smoothing. **(e)** Illustration of the Savitzky-Golay filter, making use of local polynomial fitting (dashed blue lines) to smooth the raw spatial signal (continuous black line). Regions removed by the masking are highlighted in grey. **(f)** Final spatial smoothing (red dashed line) is compared to the raw spatial signal (continuous black line).

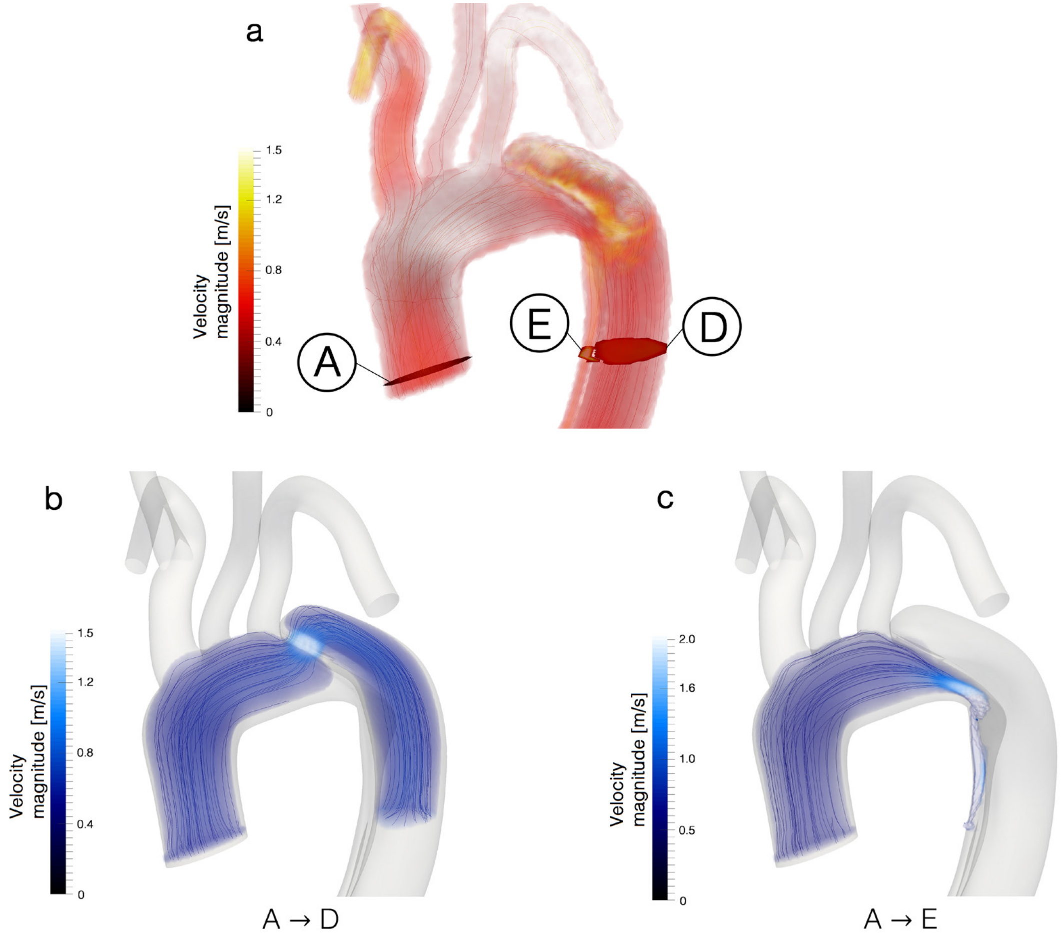

Supplementary figure 3. Visualisation of acquired and virtual flow in the dissected aortic arch. **(a)** Velocity magnitude with stream-lines of the patient-specific dissected aorta, highlighting the complex flow at the opening of the false lumen. **(b)** Velocity magnitude with stream-lines of the virtual field acquired by solving the Stokes flow boundary problem into the false lumen. **(c)** Velocity magnitude with stream lines of the virtual field acquired by solving the Stokes flow boundary problem into the true lumen.

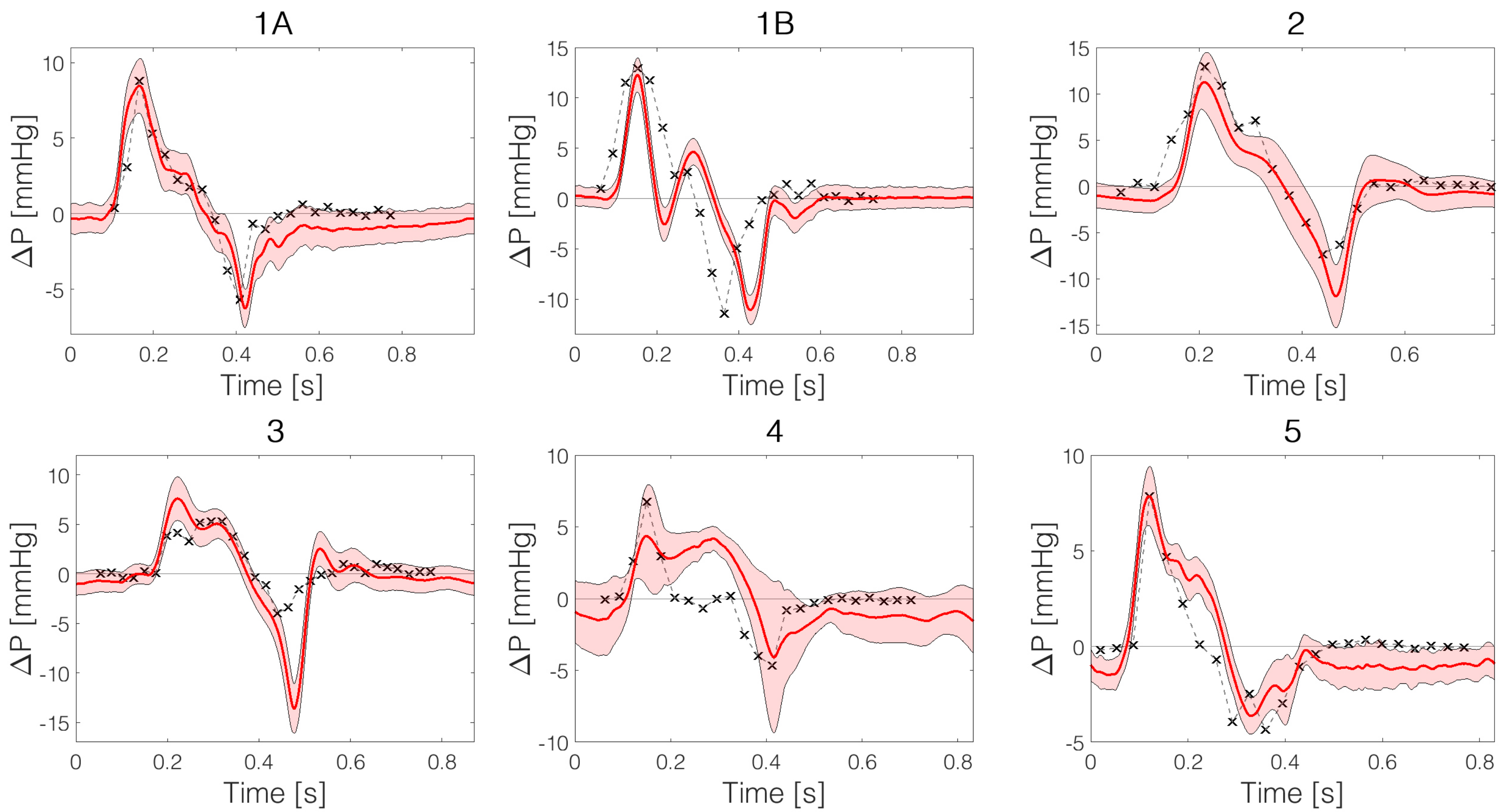

Supplementary figure 4. Validation of *v*WERP against invasive catheterisation on a cohort of patients with complex congenital heart disease. Relative pressure in the aortic arch shown from catheterisation (continuous red line, with corresponding distribution) and *v*WERP estimates (black glyphs with connecting grey dashed line). Results shown for all subjects (1A, 1B, 2-5).

Supplementary table 1. Patient characteristics for the validation study. Details given for patient age, weight, height, body surface area, sex, heart rate, systolic and diastolic blood pressure (BP) measured in the right femoral artery (RFA), as well as the base diagnosis and intervention performed. Abbreviations: Hypoplastic Left Heart Syndrome (HLHS), Tricuspid Valve (TV), Atrial Septal Defect (ASD), Ventricular Septal Defect (VSD), Partial Anomalous Pulmonary Venous Drainage (PAPVD), and Total Cavo-Pulmonary Connection (TCPC).

	Patient				
	1	*2*	*3*	*4*	*5*
Age [yrs]	10	13	9	10	14
Weight [kg]	34	25	36	47	32
Height [cm]	135	132	131	147	101
Body surface area [m^2]	1.13	0.97	1.13	1.38	0.95
Sex	M	F	M	F	M
Heart rate [bpm]	91	81	73	77	82
RFA Systolic BP [mmHg]	76	64	97	88	98
RFA Diastolic BP [mmHg]	40	36	53	52	49
Diagnosis	HLHS	HLHS	HLHS	Ebstein's anomaly of TV	Left atrial isomerism, ASD, VSD, PAPVD
Intervention	TCPC	TCPC	TCPC, LPA stent	Surgical cone procedure of TV	ASD,VSD closure, PAPVD repair

Supplementary table 2. Spatiotemporal convergence analysis in analytic setups of a laminar, steady-state, viscous flow, and a pulsatile kinetic pulsatile flow, respectively. Data is provided at spatial samplings from 4 to 1 mm^3, with temporal samplings from 8 to 64 times throughout the investigated cycle. Provided values represent the mean error to the analytic true in a noise-free, low- and high-noise case, respectively (SNR = ∞/30/10).

		$4\ mm^3$	$3\ mm^3$	$2\ mm^3$	$1\ mm^3$
SNR = ∞	*Steady-state*	10.9	12.6	11.1	3.6
	$T/8$	54.7	53.0	51.3	46.9
	$T/16$	24.0	22.6	21.3	18.5
	$T/32$	9.0	7.7	6.6	5.2
	$T/64$	6.3	5.2	4.5	4.7
SNR = 30	*Steady-state*	47.2	40.1	25.3	12.3
	$T/8$	55.2	53.7	51.4	47.3
	$T/16$	23.9	23.2	21.0	17.9
	$T/32$	9.2	8.0	6.8	5.2
	$T/64$	6.5	5.6	4.7	4.5
SNR = 10	*Steady-state*	68.4	67.0	49.7	25.9
	$T/8$	55.2	53.7	51.4	47.4
	$T/16$	24.0	23.2	21.0	17.9
	$T/32$	9.5	8.1	6.8	5.3
	$T/64$	7.6	6.2	4.8	4.6

Supplementary table 3. Performance of νWERP against alternative methods for non-invasive relative pressure assessment, specifically given for Simplified Bernoulli (SB), Unsteady Bernoulli (UB), and Work-Energy Relative Pressure (WERP), respectively. Mean error ($\varepsilon_{\Delta p}$) and mean error at maximum relative pressure ($\varepsilon_{\Delta p_{max}}$) is given at SNR = ∞ for both patient-specific *in-silico* models (aortic coarctation, CoA, and aortic dissection (AAD), respectively). The plane definitions correspond to the ones provided in Figure 3 and 4, respectively.

		SB		UB		WERP		νWERP	
Model	Plane	$\varepsilon_{\Delta p}$	$\varepsilon_{\Delta p_{max}}$	$\varepsilon_{\Delta p}$	$\varepsilon_{\Delta p_{max}}$	$\varepsilon_{\Delta p}$	$\varepsilon_{\Delta p_{max}}$	$\varepsilon_{\Delta p}$	$\varepsilon_{\Delta p_{max}}$
CoA	A → B	175.4	45.7	167.0	41.5	9865.7	27.7	10.5	7.4
	A → C	159.7	41.2	158.9	41.6	12313.3	18.8	12.8	5.0
	A → D	57.5	4.4	57.2	4.4	7457.8	21.2	5.2	0.6
	A → E	60.9	18.8	60.5	18.6	6928.0	27.1	12.6	3.2
	A → F	70.9	22.8	70.3	22.4	6623.5	24.3	10.4	0.6
	A → G	79.9	67.9	54.5	32.5	4147.0	57.4	11.1	1.4
AAD	A → B	136.3	19.6	136.1	19.6	555.2	229.5	17.2	19.6
	A → C	99.3	95.7	68.4	62.6	98.6	37.0	23.1	9.4
	A → D	86.7	84.8	71.1	4.32	176.0	48.3	10.5	10.1
	A → E	81.3	76.3	106.0	59.5	212.0	7.0	16.1	1.6
	A → F	92.8	91.1	63.2	27.1	128.7	42.1	5.0	4.9
	A → G	93.4	89.7	47.7	22.8	214.5	33.7	12.8	1.7
